# COVID-19 Pandemic Exposure and Toddler Behavioral Health in the ECHO Program

**DOI:** 10.1001/jamanetworkopen.2025.30346

**Published:** 2025-09-03

**Authors:** Anahid Akbaryan, Marie L. Churchill, Monica McGrath, Akram Alshawabkeh, Michelle Bosquet Enlow, Patricia A. Brennan, Julianna Collazo Vargas, Lauren A. Costello, Viren D’Sa, Anne Dunlop, Amy J. Elliott, Morgan Firestein, Akhgar Ghassabian, Julie A. Hofheimer, Daphne Koinis-Mitchell, Amy Margolis, Santiago Morales, Rachel Morello-Frosch, Sara S. Nozadi, Thomas G. O’Connor, Susan L. Schantz, Tracey Woodruff, Rosalind J. Wright, Lauren C. Shuffrey

**Affiliations:** 1 Department of Child and Adolescent Psychiatry, NYU Grossman School of Medicine, New York, New York; 2 Johns Hopkins Bloomberg School of Public Health, Baltimore, Maryland; 3 Department of Civil and Environmental Engineering, Northeastern University, Boston, Massachusetts; 4 Boston Children’s Hospital, Harvard Medical School, Boston, Massachusetts; 5 Department of Psychology, Emory University, Atlanta, Georgia; 6 Department of Pediatrics, Rhode Island Hospital/Alpert Medical School of Brown University, Providence; 7 Avera Research Institute, Sioux Falls, South Dakota; 8 Columbia University Medical Center, New York, New York; 9 Department of Pediatrics and Department of Population Health, NYU Grossman School of Medicine, New York, New York; 10 Division of Neonatal-Perinatal Medicine, Department of Pediatrics, University of North Carolina at Chapel Hill; 11 Department of Psychiatry and Behavioral Health, The Ohio State University, Columbus; 12 Department of Psychology, University of Southern California, Los Angeles; 13 Department of Environmental Science, Policy and Management and School of Public Health, University of California, Berkeley; 14 Community Environmental Health Program, Health Sciences Center, University of New Mexico, Albuquerque; 15 Departments of Psychiatry, Neuroscience, Obstetrics and Gynecology, University of Rochester, Rochester, New York; 16 Beckman Institute for Advanced Science and Technology and Department of Comparative Biosciences, University of Illinois, Champaign; 17 Program on Reproductive Health and the Environment, University of California, San Francisco; 18 Icahn School of Medicine at Mount Sinai, New York, New York

## Abstract

**Importance:**

Studies suggest developmental concerns for infants born during the COVID-19 pandemic, but evidence on its impact on toddler behavioral and emotional well-being remains limited.

**Objective:**

To assess whether birth timing relative to the COVID-19 pandemic is associated with toddler internalizing and externalizing problems.

**Design, Setting, and Participants:**

This retrospective cohort study utilized Environmental Influences on Child Health Outcomes (ECHO) cohort data collected between September 27, 2009, and July 21, 2023. Children were divided into 3 groups: the prepandemic group, who were born and assessed before March 13, 2020; the pandemic-assessed group, who were born before March 13, 2020, but assessed after that date; and the pandemic-born group, who were born and assessed on or after March 13, 2020. Data were collected from 9 ECHO cohort sites across the United States and Puerto Rico.

**Exposure:**

The COVID-19 pandemic, designated as starting on March 13, 2020.

**Main Outcome and Measure:**

Parent-reported internalizing and externalizing symptoms on the Preschool Child Behavior Checklist (CBCL 1½-5) at age 18 to 39 months.

**Results:**

The 3438 children (mean [SD] age, 2.33 years [5.38 months]; 1770 [51.5%] male; 537 [16.2%] Black, 1722 [50.1%] Hispanic; and 1538 [44.7%] White) were divided into 3 groups: 1323 in the prepandemic group (mean [SD] age, 2.41 years [5.66 months]); 1690 in the pandemic-assessed group (mean [SD] age, 2.32 years [5.16 months]); and 425 in the pandemic-born group (mean [SD] age, 2.14 years [4.47 months]). Both the pandemic-assessed group (unadjusted β = −1.51; 95% CI, −2.27 to −0.75; adjusted β = −1.73; 95% CI, −2.48 to −0.99) and the pandemic-born group (unadjusted β = −2.03; 95% CI, −3.13 to −0.93; adjusted β = −1.90; 95% CI, −2.99 to −0.80) had lower levels of internalizing problems compared with the prepandemic (ie, historical) group. Similarly, both the pandemic-assessed (unadjusted β = −1.74; 95% CI, −2.46 to −1.02; adjusted β = −1.81; 95% CI, −2.53 to −1.09) and the pandemic-born group (unadjusted β = −3.16; 95% CI, −4.20 to −2.12; adjusted β = −3.17; 95% CI, −4.22 to −2.12) each had lower levels of externalizing problems compared with the prepandemic group.

**Conclusions and Relevance:**

In this study, toddlers with prenatal and postnatal as well as those with only postnatal COVID-19 pandemic exposure showed fewer internalizing and externalizing problems than those born and assessed prior to the onset of the pandemic. These findings underscore the need for further research to identify protective factors that may buffer the impact of the pandemic on child behavior.

## Key Points

**Question:**

Are toddlers who experienced the COVID-19 pandemic at increased risk for internalizing and externalizing problems?

**Findings:**

This cohort study of 3438 toddlers across the United States and Puerto Rico found that those born before the pandemic but assessed during it as well as those born and assessed during the pandemic exhibited fewer parent-reported internalizing and externalizing problems compared with toddlers born and assessed before the pandemic.

**Meaning:**

These findings suggest that pandemic exposure was not associated with increased behavioral difficulties in toddlers and underscore the need to identify protective factors.

## Teaser

This cohort study evaluates whether the COVID-19 pandemic was associated with parent-reported internalizing and externalizing problems among US toddlers who participated in the Environmental Influences on Child Health Outcomes (ECHO) program.

## Introduction

The COVID-19 pandemic has profoundly impacted family dynamics and broader environmental spheres, which are crucial for children’s neurodevelopment.^1,2,3^ Early childhood is a period of heightened brain plasticity and sensitivity to environmental influences, making the early social environment pivotal for children’s health and well-being.^4,5^ Concern about child behavioral health during the COVID-19 pandemic stems from numerous studies linking the pandemic to increased levels of prenatal and postnatal maternal stress,^6,7,8,9,10,11,12,13,14^ family disruption,^3,15,16,17^ and financial difficulties,^18,19,20^ all of which have been previously associated with increased risk for adverse child behavioral outcomes.^21,22,23,24,25^

While previous research has documented increased levels of stress within the family system during the pandemic,^3,6,7,8,9,10,11,12,13,14,15,16,17,18,19,20^ findings on children’s behavioral outcomes have been mixed. Despite widespread concerns, some studies observed that children with preexisting mental health problems showed improvements during the pandemic.^26,27,28^ Global research on the effects of the pandemic on toddler developmental outcomes has also been mixed. Some studies suggest that children born during the pandemic may be at increased risk for communication, fine motor, and socioemotional delays,^29,30,31,32^ although not all studies found significant differences across developmental domains or ages.^30,31,33,34^ For example, 1 study^35^ found that pandemic-exposed children had higher problem-solving and fine motor scores at age 2 years and better cognitive performance at age 4 years but lower personal-social skills at both time points. Additionally, pandemic-related hardships were commonly reported by low-income and racial and ethnic minority families, but these stressors did not fully explain within-child changes in developmental outcomes.^36^ Notably, despite increased maternal depressive symptoms and pandemic-related challenges, some studies found minimal or no significant differences in internalizing or externalizing problems between children born before and during the pandemic after adjusting for covariates,^33,37^ highlighting the complexity of factors influencing child behavioral outcomes in this context.

Despite some research indicating associations between COVID-19 pandemic exposure and increased risk for delays in developmental milestones and socioemotional challenges,^29,30,31,32,35^ the broader implications for toddlers’ behavioral and emotional well-being remain unclear. This study examined the association between COVID-19 pandemic exposure and behavioral outcomes in toddler-aged children using data from the Environmental Influences on Child Health Outcomes (ECHO) program, which includes a geographically and sociodemographically diverse sample across multiple US regions and Puerto Rico. Behavioral outcomes were measured with the Preschool Child Behavior Checklist for Ages 1½-5 (CBCL 1½-5), a widely used and validated caregiver-report tool for assessing emotional and behavioral problems in young children.^38^ Given the well-documented negative effects of the pandemic on family functioning and parental mental health,^2,3,7,15,16,17,39,40,41^ we hypothesized that toddlers with prenatal and/or postnatal exposure to the pandemic would exhibit increased internalizing and externalizing problems compared with children born and assessed before the onset of the pandemic. This hypothesis assumed that pandemic-related stressors, such as family disruption, social isolation, and increased family stress, would contribute to more adverse neurobehavioral functioning in children.

## Methods

### ECHO Cohort

The ECHO Cohort is a National Institutes of Health–funded research consortium consisting of 69 pediatric cohort sites across the United States.^42^ The consortium investigates the associations of early-life environmental exposures with child health and development, focusing on 5 key areas: (1) prenatal, perinatal, and postnatal health; (2) obesity; (3) respiratory conditions; (4) neurodevelopment; and (5) positive health and well-being.^43,44^ Data collected under cohort-specific protocols prior to the ECHO program initiation and new data collected using the ECHO cohort data collection protocol^45^ were harmonized by the ECHO Data Analysis Center. Cohort-specific and/or central ECHO institutional review boards approved the protocols, and participants provided written and informed consent. This study followed the Strengthening the Reporting of Observational Studies in Epidemiology (STROBE) guideline for cohort studies.

### Study Population

This retrospective cohort study included ECHO participants with CBCL 1½-5 data^38^ collected between age 18 and 39 months (3438 children), with assessments conducted between August 14, 2012, and July 21, 2023. A flowchart of our sample selection is shown in the Figure. In brief, this analysis included cohorts with both a prepandemic group and at least 1 of the pandemic-exposed groups. For the prepandemic and pandemic-assessed groups, we required that the child’s age at CBCL 1½-5 assessment be 39 months or younger, which was the maximum age in the pandemic-born group. Exclusion criteria at the participant level consisted of multiple gestation, and at the cohort level, it consisted of sites that only recruited from neonatal intensive care units at birth, adoption cohort sites, or sites enriched for autism spectrum disorder. We further excluded cohort sites without CBCL 1½-5 data collected both prior to and during the COVID-19 pandemic (defined as March 13, 2020, when the president of the United States declared a COVID-19–related emergency^46^) to ensure that each included cohort contributed data to the prepandemic group and at least 1 of the pandemic-exposed groups, minimizing confounding due to site-level differences throughout the pandemic. Specific inclusion and exclusion criteria for each cohort can be found in eTable 1 in Supplement 1.

**Figure.**
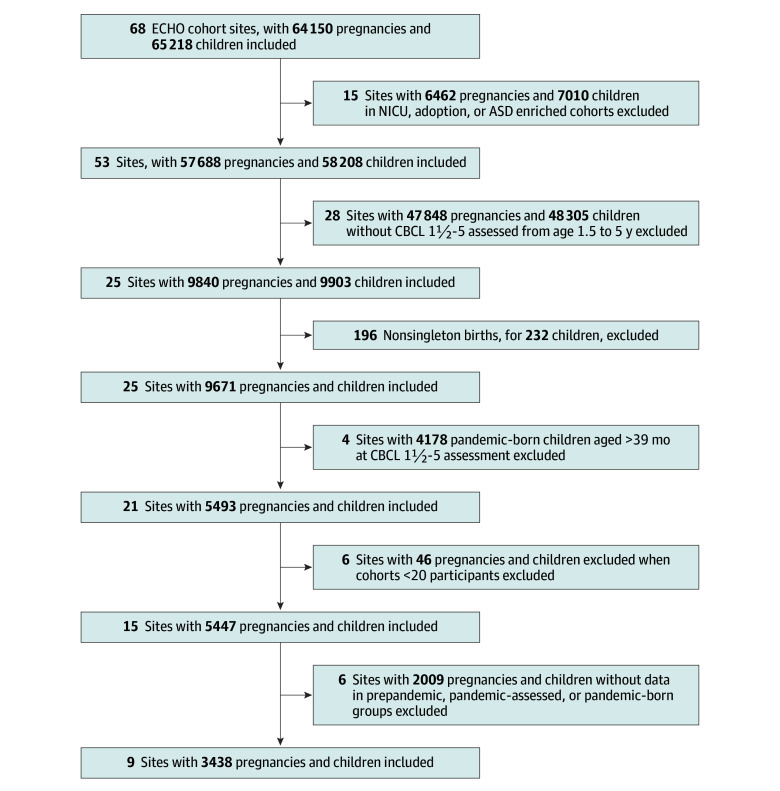
Flowchart of Study Sample Selection ASD indicates autism spectrum disorder; CBCL 1½-5, Preschool Child Behavior Checklist for Ages 1½-5; ECHO, Environmental Influences on Child Health Outcomes; NICU, neonatal intensive care unit.

### Pandemic Exposure Group Categorization

Participants were categorized based on their date of birth relative to the onset of the COVID-19 pandemic (defined as March 13, 2020^46^) and the timing of their CBCL 1½-5 assessment. Three exposure groups were defined: (1) prepandemic (ie, historical) group: children born and assessed via the CBCL 1½-5 prior to March 13, 2020; (2) pandemic-assessed group: children born prior to March 13, 2020, and assessed via the CBCL 1½-5 from March 13, 2020, to August 31, 2023; and (3) pandemic-born group: children born and assessed via the CBCL 1½-5 from March 13, 2020, to August 31, 2023 (last date of data available at time of analysis) (eFigure 1 in Supplement 1).

### CBCL 1½-5

The CBCL 1½-5 (ASEBA, licensed by the Measurement Core) is a widely used parent-report measure of child social, emotional, and behavioral concerns.^38^ It consists of 99 items that describe children’s behaviors, such as “Doesn’t get along well with other children.” For each item, caregivers are asked to describe their child’s behavior now or within the past 2 months. All items are rated on a 3-point Likert scale where 0 indicates “Not True (as far as you know)”; 1, “Somewhat or Sometimes True”; and 2, “Very True or Often True.” The original factor analysis of the CBCL 1½-5 identified 7 first-order factors, commonly referred to as syndrome scales (emotionally reactive, anxious/depressed, somatic complaints, withdrawn, sleep problems, attention problems, and aggressive behaviors) and 2 second-order factors, commonly referred to as broadband scales (internalizing problems and externalizing problems).^38^ This analysis focused on second-order factors as they are strongly associated with elevated risk for future psychological difficulties.^47^ We utilized *T* scores for internalizing and externalizing problems. When multiple CBCL 1½-5 observations were available, the most recent observation that met this age criterion was selected for analysis.

### Maternal and Child Sociodemographic and Medical History

Maternal and child sociodemographic and medical history demographic characteristics as most recently recorded prior to CBCL 1½-5 administration were harmonized across ECHO cohort sites and used in this analysis (eMethods in Supplement 1). Maternal and child race and ethnicity were self-reported. Race options included American Indian or Alaska Native, Asian, Black, Native Hawaiian or Other Pacific Islander, White, Multiple, or other. Ethnicity was reported as Hispanic or non-Hispanic.

### Statistical Analyses

Statistical analyses were conducted using R software version 4.3.3 (R Project for Statistical Computing). Means and SDs for continuous variables and frequencies and percentages for categorical variables were calculated for the overall sample and each exposure group. To examine potential differences between exposure groups, one-way analysis of variance tests were used for continuous variables, and Pearson χ^2^ tests were used for categorical variables. Linear mixed-effects models were implemented to examine the association of pandemic exposure (3-level variable: prepandemic group, pandemic-assessed group, and pandemic-born group) with toddler behavior. The primary outcomes were toddler internalizing and externalizing problems, assessed using continuous *T* scores from the CBCL 1½-5 broadband scales. All models included random intercepts for ECHO cohort site and maternal family memberships to account for correlations within sites and between siblings (153 sibling pairs) in the same family. Adjusted models included maternal race, ethnicity, education, and insurance status as well as child sex, age at CBCL 1½-5 assessment, and preterm birth as covariates. We chose to control for race and ethnicity in this analysis due to prior literature suggesting that the pandemic disproportionally affected minoritized racial and ethnic communities in the United States, including higher mortality rates^48^ and greater self-reported instrumental and financial disruptions.^49^

Pairwise comparisons were probed through linear combinations (ie, pandemic-born group vs pandemic-assessed group) using the glht command from the multcomp R package.^50^ As a sensitivity analysis, all models and comparisons were reestimated with each cohort site excluded one at a time to ensure that no single site was driving the results (eTables 2 and 3 in Supplement 1). All statistical tests were 2-sided, and a significance level of α = .05 was used. As a post hoc analysis, models were stratified by maternal educational attainment (Bachelor’s degree or higher vs less than a Bachelor’s degree) to examine potential effect modification, given emerging research suggesting that lower maternal education levels may have served as a protective factor during the pandemic,^51,52^ despite typically being associated with poorer youth mental health outcomes in nonpandemic contexts.^53,54^ Missing covariate data were imputed using the multiple imputation by chained equations from the mice R package^55^ (eMethods in Supplement 1). We report unadjusted and adjusted β estimates with 95% CIs, along with marginal and conditional *R*^2^ values, reflecting variance explained by fixed effects alone and by both fixed and random effects, respectively.

## Results

### Sample Characteristics

The sample consisted of 3438 children from 9 ECHO cohort sites enrolled across 6 US states and Puerto Rico (Table 1). The mean (SD) age of CBCL 1½-5 assessment was 2.33 years (5.38 months). Of these children, 1770 (51.5%) were assigned male sex at birth. Based on parent report, 168 (4.9%) were Asian, 537 (15.6%) were Black, 1538 (44.7%) were White, 508 (14.8%) were reported as another race, 345 (10.0%) were multiracial, and 1722 (50.1%) were Hispanic. Mothers in this sample were similarly racially and ethnically diverse (Table 1). Most mothers (1798 [52.3%]) reported having a Bachelor’s degree or greater, and 1761 (51.2%) reported either no insurance or public insurance. In total, 1323 children (38.5%; mean [SD] age at assessment, 2.41 years [5.66 months]) were included in the prepandemic group, 1690 (49.2%; mean [SD] age at assessment, 2.32 years [5.16 months]) were included in the pandemic-assessed group, and 425 (12.4%; mean [SD] age at assessment, 2.14 years [4.47 months]) were included in the pandemic-born group. The 3 exposure groups differed across several demographic and site-level characteristics (Table 1). *T* scores for all CBCL 1½–5 scales are provided (eTable 4 in Supplement 1), along with timing of the CBCL 1½–5 assessment by ECHO cohort and pandemic group (eFigure 2 in Supplement 1) and internalizing and externalizing *T* scores across these groups (eTable 5 in Supplement 1).

**Table 1.** Participant Demographic Information by Pandemic Exposure Group

Variable	Participants, No. (%)^a^				*P* value^b^
	Prepandemic group (n = 1323)	Pandemic-assessed group (n = 1690)	Pandemic-born group (n = 425)	Overall (N = 3438)	
**Maternal characteristics**					
Race					
American Indian or Alaskan Native	<15	22 (1.3)	<5	34 (1.0)	<.001
Asian	82 (6.2)	123 (7.3)	20 (4.7)	225 (6.5)	
Black	291 (22.0)	221 (13.1)	54 (12.7)	566 (16.5)	
Native Hawaiian or Other Pacific Islander	<15	28 (1.7)	<10	47 (1.4)	
White	583 (44.1)	725 (42.9)	172 (40.5)	1480 (43)	
Other	82 (6.2)	261 (15.4)	97 (22.8)	440 (12.8)	
Multiple	62 (4.7)	81 (4.8)	20 (4.7)	163 (4.7)	
Missing	201 (15.2)	229 (13.6)	53 (12.5)	483 (14.0)	
Ethnicity					
Hispanic	462 (34.9)	900 (53.3)	268 (63.1)	1630 (47.4)	<.001
Non-Hispanic	809 (61.1)	787 (46.6)	156 (36.7)	1752 (51.0)	
Missing	52 (3.9)	<5	<5	56 (1.6)	
Highest education					
Less than high school	71 (5.4)	137 (8.1)	33 (7.8)	241 (7.0)	<.001
High school degree or equivalent	181 (13.7)	320 (18.9)	89 (20.9)	590 (17.2)	
Some college, no degree	286 (21.6)	345 (20.4)	93 (21.9)	724 (21.1)	
Bachelor’s degree and above	751 (56.8)	843 (49.9)	204 (48.0)	1798 (52.3)	
Missing	34 (2.6)	45 (2.7)	6 (1.4)	85 (2.5)	
Last known public insurance^c^					
No insurance or public insurance	613 (46.3)	928 (54.9)	220 (51.8)	1761 (51.2)	.65
Private or other insurance	457 (34.5)	710 (42.0)	183 (43.1)	1350 (39.3)	
Missing	253 (19.1)	52 (3.1)	22 (5.2)	327 (9.5)	
Last known household income, $^c^					
<30 000	317 (24.0)	494 (29.2)	139 (32.7)	950 (27.6)	.02
30 000-$49 999	141 (10.7)	193 (11.4)	49 (11.5)	383 (11.1)	
50 000-$74 999	123 (9.3)	141 (8.3)	30 (7.1)	294 (8.6)	
75 000-$99 999	82 (6.2)	93 (5.5)	31 (7.3)	206 (6.0)	
100 000-$199 999	193 (14.6)	220 (13.0)	53 (12.5)	466 (13.6)	
≥200 000	201 (15.2)	297 (17.6)	56 (13.2)	554 (16.1)	
Missing	266 (20.1)	252 (14.9)	67 (15.8)	585 (17.0)	
**Child characteristics**					
Sex at birth					
Male	663 (50.1)	884 (52.3)	223 (52.5)	1770 (51.5)	.44
Female	660 (49.9)	806 (47.7)	202 (47.5)	1668 (48.5)	
Missing	0	0	0	0	
Race					
American Indian or Alaskan Native	17 (1.3)	40 (2.4)	10 (2.4)	67 (1.9)	<.001
Asian	55 (4.2)	102 (6.0)	11 (2.6)	168 (4.9)	
Black	274 (20.7)	216 (12.8)	47 (11.1)	537 (15.6)	
Native Hawaiian or Other Pacific Islander	5 (0.4)	19 (1.1)	14 (3.3)	38 (1.1)	
White	606 (45.8)	747 (44.2)	185 (43.5)	1538 (44.7)	
Other	129 (9.8)	324 (19.2)	55 (12.9)	508 (14.8)	
Multiple	152 (11.5)	164 (9.7)	29 (6.8)	345 (10.0)	
Missing	85 (6.4)	78 (4.6)	74 (17.4)	237 (6.9)	
Ethnicity					
Hispanic	517 (39.1)	941 (55.7)	264 (62.1)	1722 (50.1)	<.001
Non-Hispanic	795 (60.1)	726 (43)	119 (28.0)	1640 (47.7)	
Missing	11 (0.8)	23 (1.4)	42 (9.9)	76 (2.2)	
Gestational age at birth, completed wk					
Mean (SD)	38.68 (2.01)	38.71 (1.75)	38.59 (1.67)	38.68 (1.84)	.49
Median (range)	39.00 (24.00-43.00)	39.00 (23.00-42.00)	39.00 (26.00-43.00)	39.00 (23.00-43.00)	
Missing, No. (%)	6 (0.5)	<5	<5	12 (0.3)	
**CBCL 1½-5 Outcomes**					
Child age at CBCL 1½-5 assessment, mo					
Mean (SD)	28.96 (5.66)	27.78 (5.16)	25.62 (4.47)	27.97 (5.38)	<.001
Median (range)	26.77 (18.17-38.96)	25.00 (18.00-39.00)	24.00 (18.00-39.00)	25.00 (18.00-39.00)	
Missing, No.	0	0	0	0	
CBCL internalizing problem *T* score					
Mean (SD)	44.12 (10.19)	42.29 (10.05)	42.02 (10.31)	42.96 (10.17)	<.001
Median (range)	43.00 (29.00-74.00)	41.00 (29.00-81.00)	41.00 (29.00-73.00)	43.00 (29.00-81.00)	
Missing, No.	0	0	0	0	
CBCL 1½-5 internalizing problems clinical ranges					
Normal range (≤59)	1201 (90.8)	1582 (93.6)	395 (92.9)	3178 (92.4)	.05
Borderline clinical range (≥60 to ≤63)	73 (5.5)	58 (3.4)	17 (4.0)	148 (4.3)	
Clinical range (≥64)	49 (3.7)	50 (3.0)	13 (3.1)	112 (3.3)	
Missing	0	0	0	0	
CBCL 1½-5 externalizing problems *T* score					
Mean (SD)	45.73 (9.78)	43.71 (9.52)	42.68 (9.65)	44.36 (9.70)	<.001
Median (range)	46.00 (28.00-79.00)	43.00 (28.00-77.00)	42.00 (28.00-73.00)	43.00 (28.00-79.00)	
Missing, No.	0	0	0	0	
CBCL 1½-5 externalizing problems clinical ranges					
Normal range (≤59)	1207 (91.2)	1590 (94.1)	403 (94.8)	3200 (93.1)	.01
Borderline clinical range (≥60 to ≤63)	65 (4.9)	58 (3.4)	10 (2.4)	133 (3.9)	
Clinical range (≥64)	51 (3.9)	42 (2.5)	12 (2.8)	105 (3.1)	
Missing	0	0	0	0	
**Cohort characteristics^d^**					
State of cohort site, No. (%)					
CA	159 (12.0)	174 (10.3)	13 (3.1)	346 (10.1)	<.001
GA	163 (12.3)	98 (5.8)	14 (3.3)	275 (8.0)	
IL	215 (16.3)	100 (5.9)	14 (3.3)	329 (9.6)	
MA	<5	0	0	<5	
NY	508 (38.4)	996 (58.9)	264 (62.1)	1768 (51.4)	
PR	139 (10.5)	309 (18.3)	107 (25.2)	555 (16.1)	
RI	<140	13 (0.8)	13 (3.1)	<165	
Missing	0	0	0	0	
Cohort site					
PROTECT	89 (6.7)	229 (13.6)	96 (22.6)	414 (12.0)	<.001
BAMBAM	137 (10.4)	13 (0.8)	13 (3.1)	163 (4.7)	
BYS	93 (7.0)	133 (7.9)	30 (7.1)	256 (7.4)	
Atlanta ECHO Cohort of Emory University	163 (12.3)	98 (5.8)	14 (3.3)	275 (8.0)	
Fair Start	43 (3.3)	16 (0.9)	34 (8.0)	93 (2.7)	
IKIDS	215 (16.3)	100 (5.9)	14 (3.3)	329 (9.6)	
CIOB	159 (12.0)	174 (10.3)	13 (3.1)	346 (10.1)	
NYU CHES	297 (22.4)	844 (49.9)	163 (38.4)	1304 (37.9)	
PRISM	127 (9.6)	83 (4.9)	48 (11.3)	258 (7.5)	
Missing	0	0	<5	0	

Abbreviations: BAMBAM, Behavior and Mood in Babies and Mothers; BYS, Boricua Youth Study; CBCL 1½-5, Child Behavior Checklist for Ages 1½-5; CIOB, Chemicals in our Bodies; ECHO, Environmental influences on Child Health Outcomes; IKIDS, Illinois Kids Development Study; NYU CHES, NYU Children’s Health and Environment Study; PRISM, Programming of Intergenerational Stress Mechanisms; PROTECT, ECHO in Puerto Rico.

^a^ In accordance with ECHO’s publication and data usage policy, any cells with values smaller than 5 are suppressed.

^b^ *P* values are from Pearson χ^2^ tests for categorical variables and from analysis of variance tests for continuous variables.

^c^ Among participants with data.

^d^ Cohort site state does not necessarily reflect where the participant resided at the time of recruitment or follow-up.

### Pandemic Exposure Group and Child Internalizing Problems

There was a significant association of pandemic exposure group with child internalizing problem *T* scores (unadjusted model: marginal *R*^2^ = 0.006; conditional *R*^2^ = 0.434; adjusted model: marginal *R*^2^ = 0.065; conditional *R*^2^ = 0.430; pooled likelihood ratio, F_14,28 168.53_ = 11.45; *P* < .001) (Table 2). Compared with the prepandemic group, children in the pandemic-assessed group had internalizing problem *T* scores that were 1.5 points lower in unadjusted models (β = −1.51; 95% CI, −2.27 to −0.75) and 1.7 points lower in adjusted models (β = −1.74; 95% CI, −2.48 to −0.99). Similarly, children in the pandemic-born group had internalizing *T* scores that were 2.0 points lower in unadjusted models (β = −2.03; 95% CI, −3.13 to −0.93), and 1.9 points lower in adjusted models (β = −1.90; 95% CI, −2.99 to −0.80) compared with the prepandemic group. In post hoc contrast testing, internalizing problem *T* scores did not significantly differ between the 2 pandemic-exposed groups (unadjusted β = −0.54; 95% CI, −1.58 to 0.53; adjusted β = −0.15; 95% CI, −1.19 to 0.90) (Table 3). Leave-one-out analysis revealed that no single cohort site considerably affected these results (eTable 2 in Supplement 1).

**Table 2.** Association of Pandemic Exposure Group With Child Internalizing and Externalizing Problems

Group	β (95% CI)	
	Model 1^a^	Model 2^b^
Internalizing problems		
Sample size, No.	3438	3438
Marginal R^2^	0.006	0.065
Condition *R*^2^	0.434	0.430
D_3_^c^	NA	F_14,28 168.53_ = 11.45; *P* < .001
Prepandemic group	0 [Reference]	0 [Reference]
Pandemic-assessed group	−1.506 (−2.27 to −0.75)^d^	−1.735 (−2.48 to −0.99)^d^
Pandemic-born group	−2.031 (−3.13 to −0.93)^d^	−1.895 (−2.99 to −0.80)^d^
**Externalizing problems**		
Sample size, No.	3438	3438
Marginal *R*^2^	0.012	0.033
Conditional *R*^2^	0.517	0.510
D_3_^c^	NA	F_14,34 952.82_ = 4.00; *P* < .001
Prepandemic group	0 [Reference]	0 [Reference]
Pandemic-assessed goup	−1.740 (−2.46 to −1.02)^d^	−1.806 (−2.53 to −1.09)^d^
Pandemic-born group	−3.162 (−4.20 to −2.12)^d^	−3.168 (−4.22 to −2.12)^d^

Abbreviation: NA, not applicable.

^a^ Models include random intercepts for cohort site membership and maternal family.

^b^ Models include random intercepts for cohort site membership and maternal family and were adjusted for maternal race (categorical), maternal ethnicity (binary), maternal highest education (categorical), maternal insurance status (binary), child sex (binary), child age at assessment (continuous), and preterm birth status (binary).

^c^ D_3 _is the pooled likelihood ratio test statistics comparing current model 2 with model 1, with corresponding outcome.

^d^ *P* < .001.

**Table 3.** Linear Combinations of Pandemic Exposure Group With Child Internalizing and Externalizing Problems

Group	β (95% CI)	
	Model 1^a^	Model 2^b^
Internalizing problems		
Sample size, No.	3438	3438
Pandemic-assessed group	0 [Reference]	0 [Reference]
Pandemic-born group	−0.525 (−1.58 to 0.53)	−0.145 (−1.19 to 0.90)
**Externalizing problems**		
Sample size, No.	3438	3438
Pandemic-assessed group	0 [Reference]	0 [Reference]
Pandemic-born group	−1.422 (−2.41 to −0.43)^c^	−1.354 (−2.35 to −0.35)^c^

^a^ Models include random intercepts for cohort site membership and family.

^b^ Models include random intercepts for cohort site membership and maternal family and were adjusted for maternal race (categorical), maternal ethnicity (binary), maternal highest education (categorical), maternal insurance status (binary), child sex (binary), child age at assessment (continuous), and preterm birth status (binary).

^c^ *P* < .05.

Associations of the pandemic exposure group on child internalizing problems were directionally similar across maternal education strata. However, they were larger and statistically significant in both pandemic-exposed groups only in children of mothers with less than a Bachelor’s degree (Table 4; eResults in Supplement 1).

**Table 4.** Adjusted Association of Pandemic Exposure Group With Child Internalizing and Externalizing Problems, by Maternal Education Status

Group	Maternal education, β (95% CI)	
	Less than bachelor’s degree^a^	Bachelor’s degree and greater^a^
Internalizing problems		
Sample size, No.	1555	1798
Marginal *R*^2^	0.034	0.030
Condition *R*^2^	0.461	0.323
Pre-pandemic group	0 [Reference]	0 [Reference]
Pandemic-assessed group	−2.710 (−3.91 to −1.51)^b^	−1.019 (−1.98 to −0.06)^c^
Pandemic-born group	−3.906 (−5.60 to −2.21)^b^	−0.380 (−1.86 to 1.10)
**Externalizing problems**		
Sample size, No.	1555	1798
Marginal *R*^2^	0.043	0.018
Conditional *R*^2^	0.585	0.413
Pre-pandemic group	0 [Reference]	0 [Reference]
Pandemic-assessed group	−2.981 (−4.11 to −1.86)^b^	−0.949 (−1.90 to 0.00)
Pandemic-born group	−5.128 (−6.69 to −3.56)^b^	−2.064 (−3.53 to −0.60)^c^

^a^ Models include random intercepts for cohort site membership and maternal family and were adjusted for maternal race (categorical), maternal ethnicity (binary), maternal highest education (categorical), maternal insurance status (binary), child sex (binary), child age at assessment (continuous), and preterm birth status (binary).

^b^ *P* < .001.

^c^ *P* < .05.

### Pandemic Exposure Group and Child Externalizing Problems

There was a significant association of pandemic exposure group with child externalizing problem *T* scores (unadjusted model: marginal *R*^2^ = 0.012; conditional *R*^2^ = 0.517; adjusted model: marginal *R*^2^ = 0.033; conditional *R*^2^ = 0.510; pooled likelihood ratio, F_14,34 952.82_ = 4.00; *P* < .001) (Table 2). Compared with the prepandemic group, children in the pandemic-assessed group had externalizing problem *T* scores that were 1.7 points lower in unadjusted models (β = −1.74; 95% CI, −2.46 to −1.02) and 1.8 points lower in adjusted models (β = −1.81; 95% CI, −2.53 to −1.09). Children in the pandemic-born group had externalizing problem *T* scores that were 3.2 points lower in unadjusted models (β = −3.16; 95% CI, −4.20 to −2.12) and 3.2 points lower in adjusted models (β = −3.17; 95% CI, −4.22 to −2.12) compared with the prepandemic group. Furthermore, children in the pandemic-born group had significantly lower levels of externalizing problem *T* scores compared with children in the pandemic-assessed group (unadjusted β = −1.42; 95% CI, −2.41 to −0.43; adjusted β = −1.35; 95% CI, −2.35 to −0.35) (Table 3). Leave-one-out analysis revealed that no single cohort site considerably affected these results (eTable 3 in Supplement 1).

Associations of the pandemic exposure group on child externalizing problems were directionally similar across maternal education strata. However, they were larger and statistically significant in both pandemic-exposed groups only in children of mothers with less than a Bachelor’s degree (Table 4; eResults in Supplement 1).

## Discussion

This retrospective cohort study examined associations between COVID-19 pandemic exposure and toddler internalizing and externalizing problems. Contrary to our hypothesis, children in the pandemic-assessed and pandemic-born groups had lower internalizing and externalizing problem scores compared with those in the prepandemic group. These differences were more pronounced among children of mothers with less than a Bachelor’s degree compared with children of mothers with higher educational attainment.

To our knowledge, this is the first study to investigate these associations across diverse US regions and Puerto Rico among 3 distinct groups of toddler-aged children: those born and assessed prior to the pandemic, those born prior to but assessed during the pandemic, and those both born and assessed during the pandemic. This multisite, longitudinal study provided a unique opportunity to examine toddler behavioral outcomes related to the pandemic. Previous research has observed associations between the pandemic and changes in adolescent brain and behavioral development, such as cortical thinning and larger bilateral hippocampal and amygdala volume, which are patterns typically seen in individuals exposed to significant childhood adversity.^56^ However, we found no significant associations between pandemic exposure and increased child internalizing and externalizing problems. Similarly, other research reported no significant differences in internalizing and externalizing behaviors between 2-year-olds born during the pandemic and a prepandemic cohort.^33^ Another study of children and adolescents aged 5 to 17 years found no link between COVID-19–related stressors and increased internalizing and externalizing behaviors, but identified parental mental health, particularly depressive and anxiety symptoms, and negative parenting styles (eg, hostility, poor supervision) as stronger predictors.^57^

Emerging evidence suggests that the pandemic may have impacted children differently depending on family characteristics such as socioeconomic status (SES) and caregiving practices. One study found that families facing greater economic hardship may have been more proactive in alleviating emotional distress among their children,^58^ despite lower SES typically being associated with higher rates of emotional and behavioral difficulties in youth before the pandemic.^59,60,61^ Other work found greater life satisfaction declines in higher-SES families, suggesting they may have struggled more with pandemic-related disruptions.^62^ Within the home, consistent family routines, such as regular playtimes, bedtime rituals (eg, reading a story, a goodnight kiss), and family meals, have been associated with fewer depressive symptoms in preschool children during the pandemic.^63^

Our findings regarding maternal education align with those of other studies examining the pandemic’s impact on child behavioral outcomes. One study identified 2 clusters based on pandemic-related changes in health behaviors, coping strategies, and social isolation: a low-change cluster (lower educational attainment) and a high-change cluster (higher educational attainment).^51^ The low-change cluster reported fewer disruptions, lower stress, and less social isolation, while the high-change cluster experienced greater life disruptions and social isolation. Despite higher socioeconomic advantages in the high-change group, children of mothers in the low-change cluster showed fewer behavioral problems, potentially reflecting a more stable caregiving environment. Another study found mothers with some college or a 4-year degree experienced higher pandemic stress than those with the least and highest educational attainment,^52^ indicating that the relationship between education and pandemic stress is complex and that other factors likely contribute to these outcomes.

### Limitations

This study has several limitations impacting both the measurement and interpretation of child behavioral outcomes during the pandemic. First, reliance on caregiver-reported child behavior may introduce subjectivity due to parental mental health, biases, and expectations.^64,65^ The pandemic may have further influenced these perceptions, potentially leading parents to view behaviors as less concerning amid broader stressors. We also did not assess parent mental health, which could have provided important context. The narrow toddler-age range of our sample limits generalizability to older children, who may have experienced different behavioral impacts related to disrupted peer interactions, school attendance, and other social activities. Unmeasured confounders, such as changes in parenting or the home environment, may have influenced findings. Additionally, we did not examine specific COVID-19 stressors, such as health concerns, job loss, social isolation, or disrupted routines, that could influence child outcomes. We also lacked data on prenatal SARS-CoV-2 exposure, which may have subtle effects on child neurobehavior.^66^ Finally, data were pooled from 9 cohorts across 6 states and Puerto Rico, each with unique pandemic experiences, potentially contributing to regional variability in pandemic-related experiences due to local policy and other contextual factors.

## Conclusions

In this cohort study of a diverse US sample, pandemic-assessed and pandemic-born toddlers had lower internalizing and externalizing problem *T *scores than a prepandemic group. Although individual-level differences between prepandemic and pandemic-exposed groups may appear small, even modest shifts in group-level scores can signal meaningful changes in behavioral or emotional functioning, with significant public health implications, especially when sustained across large populations. These findings highlight the need for further research to identify potential protective factors that may have promoted resilience among children exposed to the COVID-19 pandemic.

## Notes

Supplement 1. **eTable 1.** Cohort Inclusion and Exclusion Criteria
eMethods.
**eFigure 1.** Timeline Displaying the Range of Birth and CBCL 1½-5 Assessment Dates Across Exposure Groups **eTable 2.** Leave One Out Sensitivity Analyses Examining the Association Between Timing of Birth and CBCL 1½-5 Assessment Relative to the COVID-19 Pandemic With Child Internalizing Behaviors **eTable 3.** Leave One Out Sensitivity Analyses Examining the Association Between Timing of Birth and CBCL 1½-5 Assessment Relative to the COVID-19 Pandemic With Child Externalizing Behaviors **eFigure 2.** CBCL 1½-5 Assessment Timing Across Cohorts and Exposure Groups **eTable 4.** Descriptive Table of All CBCL Outcomes Across Exposure Groups
eResults.
**eTable 5.** CBCL 1½-5 Internalizing and Externalizing *T* Scores Across Assessment Dates and Exposure Groups
eReferences.

Supplement 2.
Nonauthor Collaborators

Supplement 3.
Data Sharing Statement
