# Supplementary material for: COVID-19 Pandemic Exposure and Toddler Behavioral Health in the ECHO Program
Source: JAMA Netw Open. 2025 Sep 3;8(9):e2530346. doi: 10.1001/jamanetworkopen.2025.30346 (PMC12409581; doi:10.1001/jamanetworkopen.2025.30346)
Supplement: Supplement 1. — eTable 1. Cohort Inclusion and Exclusion Criteria eMethods. eFigure 1. Timeline Displaying the Range of Birth and CBCL 1½-5 Assessment Dates Across Exposure Groups eTable 2. Leave One Out Sensitivity Analyses Examining the Association Between Timing of Birth and CBCL 1½-5 Assessment Relative to the COVID-19 Pandemic With Child Internalizing Behaviors eTable 3. Leave One Out Sensitivity Analyses Examining the Association Between Timing of Birth and CBCL 1½-5 Assessment Relative to the COVID-19 Pandemic With Child Externalizing Behaviors eFigure 2. CBCL 1½-5 Assessment Timing Across Cohorts and Exposure Groups eTable 4. Descriptive Table of All CBCL Outcomes Across Exposure Groups eResults. eTable 5. CBCL 1½-5 Internalizing and Externalizing T Scores Across Assessment Dates and Exposure Groups eReferences. [file jamanetwopen-e2530346-s001.pdf]

Supplemental Online Content

Akbaryan A, Churchill ML, McGrath M, et al; ECHO Cohort Consortium. COVID-19 pandemic exposure and toddler behavioral health in the ECHO program. *JAMA Netw Open.* 2025;8(9):e2530346. doi:10.1001/jamanetworkopen.2025.30346

eTable 1. Cohort Inclusion and Exclusion Criteria

eMethods.

eFigure 1. Timeline Displaying the Range of Birth and CBCL 1½-5 Assessment Dates Across Exposure Groups

eTable 2. Leave One Out Sensitivity Analyses Examining the Association Between Timing of Birth and CBCL 1½-5 Assessment Relative to the COVID-19 Pandemic With Child Internalizing Behaviors

eTable 3. Leave One Out Sensitivity Analyses Examining the Association Between Timing of Birth and CBCL 1½-5 Assessment Relative to the COVID-19 Pandemic With Child Externalizing Behaviors

eFigure 2. CBCL 1½-5 Assessment Timing Across Cohorts and Exposure Groups

eTable 4. Descriptive Table of All CBCL Outcomes Across Exposure Groups

eResults.

eTable 5. CBCL 1½-5 Internalizing and Externalizing *T* Scores Across Assessment Dates and Exposure Groups

eReferences.

This supplemental material has been provided by the authors to give readers additional information about their work.

© 2025 Akbaryan A et al. *JAMA Network Open*.

eTable 1. Cohort Inclusion and Exclusion Criteria

Cohort Site	Inclusion Criteria	Exclusion Criteria
PROTECT	1. Pregnant woman with less than 20 weeks gestation<br>2. Planned to give birth in one of the affiliated hospitals<br>3. Seeking prenatal care in one of the affiliated clinics	1. Age less than 18 or over 40 years<br>2. Multiple fetal pregnancies<br>3. Have used contraceptive methods on the 3 months prior to pregnancy<br>4. Have used in vitro fertilization to conceive<br>5. History of threatened abortion<br>6. Major vaginal bleeding<br>7. Cardiac disease prior to pregnancy<br>8. Chronic high blood pressure<br>9. History of neuropathy<br>10. Liver disease prior to pregnancy
BAMBAM	1. Healthy children<br>2. No metal in body (MR safe for the MRI component of study)<br>3. No diagnosis of ADHD<br>4. No older than 12 years	1. Children with major risk factors for brain abnormalities<br>2. In utero alcohol, cigarette, or illicit substance exposure<br>3. Preterm (<37 wks gestation) birth<br>4. SGA or less than 1500g<br>5. Fetal ultrasound abnormalities<br>6. Complicated pregnancy including preeclampsia, high blood pressure, or GDM<br>7. APGAR scores <8<br>8. NICU admission<br>9. Neurological disorder (e.g., head injury, epilepsy)<br>10. Psychiatric or learning disorder in the infant, parents, or siblings (such as medicated depression)
BYS	1. Participation in the Boricua Youth Study, Wave 1 (BYS W1), which required:<br>2. Being between the ages of 5–13 years in 2001–2002<br>3. Living in South Bronx, New York, or the San Juan Metropolitan Area or Caguas, Puerto Rico in 2001–2002<br>4. Being of Puerto Rican background at enumeration of BYS W1<br>5. At least one of the youth's caretakers identified as Puerto Rican background	1. Youth known to be severely impaired neuropsychologically<br>2. Youth who were permanently institutionalized or youth who had run away or left home<br>3. Emancipated youth whose parent could not provide consent
ECHO Emory	1. African American (self-identified as Black/African American and self-report as US-born)<br>2. 18–40 years of age at the time of enrollment<br>3. Singleton pregnancy between 8–14 weeks<br>4. Fewer than 5 previous births<br>5. No chronic medical conditions and not taking prescription medications chronically	1. Post-enrollment: Intrauterine death<br>2. Post-enrollment: Congenital anomalies

© 2025 Akbaryan A et al. *JAMA Network Open*.

Fair Start	1. Enrollment at the Columbia Center for Family and Community Medicine clinic	1. Pregnant with >1 fetus
IKIDS	1. Pregnant women must be between 18–40 years of age<br>2. Fluent in English<br>3. Live within 30-minute drive from research lab<br>4. Prenatal care must be done at Christie or Carle OB-GYN clinics in Champaign-Urbana area<br>5. Birth of infant must happen at Presence Medical Center or Carle Foundation Hospital in Urbana<br>6. Only one child per mother may participate<br>7. Able to enroll in study and provide first urine sample by 14 weeks gestation<br>8. Able/willing to come to research lab for child assessments post delivery	1. High-risk pregnancy (including expecting multiple births)<br>2. A serious health condition involving either the child or mother during pregnancy<br>3. A health condition that prevents or limits the child's participation in postnatal assessments<br>4. Mother loses custody of child and/or is unwilling or unable to complete postnatal surveys
CIOB	1. Pregnant women between 13 and 27 weeks of gestation<br>2. Age 18+<br>3. English or Spanish speaking<br>4. Delivery at Zuckerberg San Francisco General Hospital or the UCSF Betty Irene Moore Women's Hospital at Mission<br>5. Singleton births	1. High-risk pregnancy
NYU CHES	1. At least 18 weeks pregnant<br>2. At least 18 years of age<br>3. Planned to deliver at a study hospital site (Lutheran, Bellevue or Tisch)	N/A
PRISM	1. Mother 18 years or older at recruitment in pregnancy<br>2. Single-gestation pregnancy<br>3. Mother English or Spanish speaking	1. At enrollment, endorsement of drinking ≥ 7 alcoholic drinks/week prior to pregnancy recognition<br>2. At enrollment, endorsement of any drinking after pregnancy recognition<br>3. Maternal or child chronic health conditions that would impede study participation

Abbreviations: ADHD, attention-deficit/hyperactivity disorder; APGAR, appearance, pulse, grimace, activity, and respiration; GDM, gestational diabetes mellitus; MRI, magnetic resonance imaging; NICU, neonatal intensive care unit; SGA, small for gestation ag

© 2025 Akbaryan A et al. *JAMA Network Open*.

eMethods.

Maternal and Child Sociodemographic and Medical History

The following maternal and child sociodemographic and medical history demographics, as most recently recorded prior to CBCL 1½-5 administration, were harmonized across Environmental influences on Child Health Outcomes (ECHO) cohort sites and used in this analysis: maternal race (American Indian or Alaska Native (AIAN), Asian, Black, Native Hawaiian or other Pacific Islander (PI), White, other race, or multiple races), maternal ethnicity (Hispanic, non-Hispanic), maternal highest education attained (less than high school, high school degree or equivalent, some college but no degree, or Bachelor's degree and above), last known maternal insurance (no insurance or public insurance, including Medicare or Medicaid; private, including employment-based marketplace, TRICARE, VA, and HIS, or other insurance), child sex assigned at birth (male, female), preterm birth (gestational age at birth < 37 weeks or ≥ 37 weeks), and child age at CBCL 1½-5 administration. Sociodemographic and medical history data were assessed via maternal self-report using ECHO data collection forms or through maternal-newborn medical record abstraction. Additionally, gestational age at birth and last known household income (<$30,000, $30,000–$49,999, $50,000–$74,999, $75,000–$99,999, $100,000–$199,999, ≥ $200,000) were included to further describe the sample.

Supplementary Statistical Analyses

Imputation of missing covariate data. Missing covariate data were imputed using multiple imputation by chained equations from the "mice" R package.[1] Results were pooled after 25 imputations with a maximum of 10 iterations. The imputation models included our variables of interest, with cohort-site membership as a classification variable and family as a "level-2" cluster variable for variables that are the same within a family (e.g., maternal highest education, maternal race, maternal ethnicity, maternal last known insurance status, and household income). Conditional and marginal R^2s were pooled and calculated by applying Rubin's rule and Fisher's Z transformation[2]. Pooled likelihood ratio tests were run, and D_3 statistics were calculated as $F_{k,v}$ using the "D3" function in "mice".[3]

© 2025 Akbaryan A et al. *JAMA Network Open*.

eFigure 1. Timeline Displaying the Range of Birth and CBCL 1½-5 Assessment Dates Across Exposure Groups

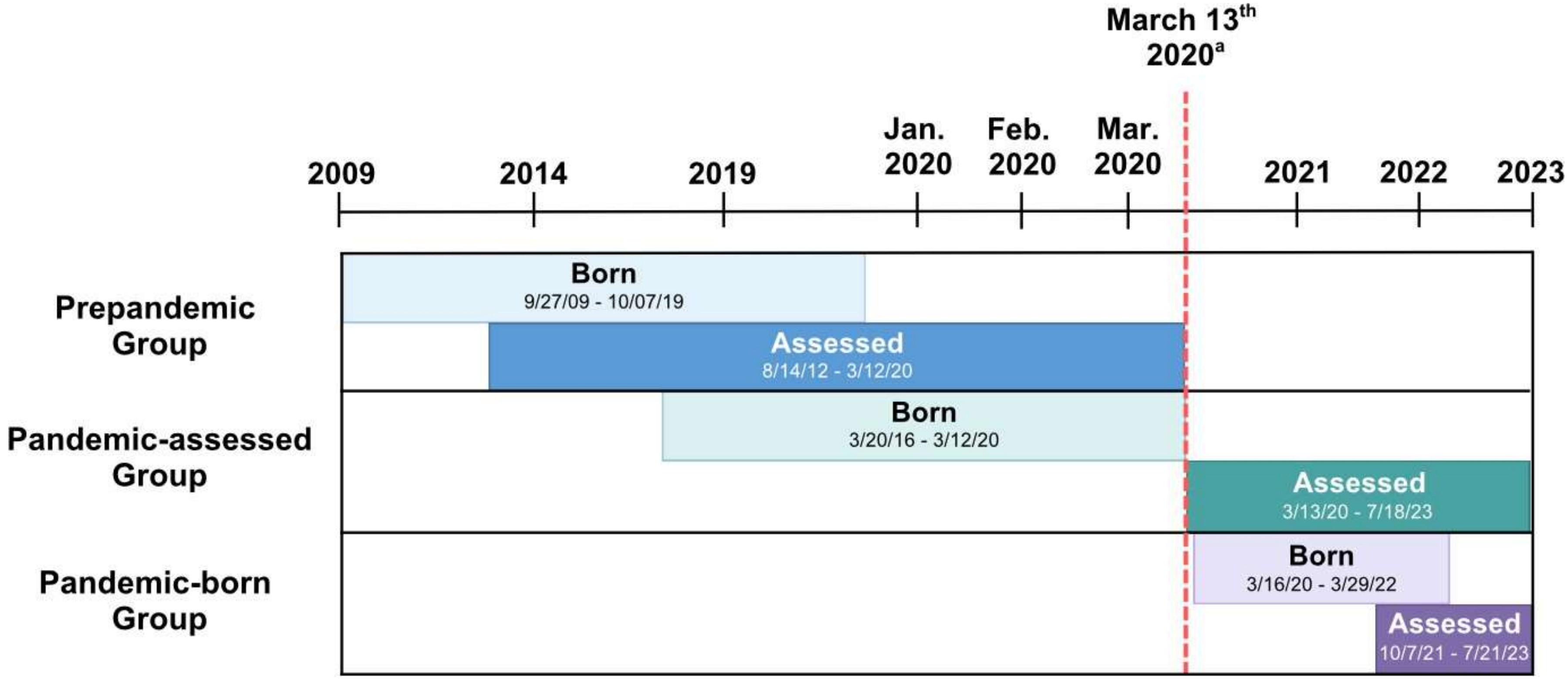

[a]Date when the President of the United States declared a COVID-related emergency.[4]

© 2025 Akbaryan A et al. *JAMA Network Open*.

eTable 2. Leave One Out Sensitivity Analyses Examining the Association Between Timing of Birth and CBCL 1½-5 Assessment Relative to the COVID-19 Pandemic With Child Internalizing Behaviors

	Model 1[a] β[95% CI]	Model 2[b] β[95% CI]
**Sample: Leave out PROTECT**		
Sample Size	*N=3024*	*N=3024*
Prepandemic Group	REF	REF
Pandemic-assessed Group	-1.479 [-2.28, -0.68]**	-1.738 [-2.53, -0.95]**
Pandemic-born Group	-2.269 [-3.48, -1.06]**	-2.036 [-3.24, -0.84]**
**Sample: Leave out BAMBAM**		
Sample Size	*N=3275*	*N=3275*
Prepandemic Group	REF	REF
Pandemic-assessed Group	-1.503 [-2.27, -0.73]**	-1.741 [-2.5, -0.98]**
Pandemic-born Group	-1.886 [-3.02, -0.76]*	-1.765 [-2.89, -0.64]*
**Sample: Leave out BYS**		
Sample Size	*N=3182*	*N=3182*
Prepandemic Group	REF	REF
Pandemic-assessed Group	-1.573 [-2.36, -0.78]**	-1.834 [-2.61, -1.06]**
Pandemic-born Group	-2.143 [-3.29, -1]**	-2.043 [-3.18, -0.91]**
**Sample: Leave out ECHO Emory**		
Sample Size	*N=3163*	*N=3163*
Prepandemic Group	REF	REF
Pandemic-assessed Group	-1.327 [-2.12, -0.53]*	-1.572 [-2.35, -0.79]**
Pandemic-born Group	-1.838 [-2.96, -0.71]*	-1.654 [-2.77, -0.54]*
**Sample: Leave out Fair Start**		
Sample Size	*N=3345*	*N=3345*
Prepandemic Group	REF	REF
Pandemic-assessed Group	-1.532 [-2.3, -0.77]**	-1.792 [-2.54, -1.04]**
Pandemic-born Group	-2.102 [-3.24, -0.97]**	-1.963 [-3.08, -0.84]**

© 2025 Akbaryan A et al. *JAMA Network Open*.

**Sample: Leave out IKIDS**		
Sample Size	*N=3109*	*N=3109*
Prepandemic Group	REF	REF
Pandemic-assessed Group	-1.566 [-2.38, -0.76]**	-1.803 [-2.6, -1.01]**
Pandemic-born Group	-2.204 [-3.35, -1.06]**	-2.086 [-3.22, -0.95]**
**Sample: Leave out CIOB**		
Sample Size	*N=3092*	*N=3092*
Prepandemic Group	REF	REF
Pandemic-assessed Group	-1.681 [-2.5, -0.86]**	-1.875 [-2.68, -1.07]**
Pandemic-born Group	-2.216 [-3.36, -1.08]**	-2.053 [-3.19, -0.92]**
**Sample: Leave out NYU CHES**		
Sample Size	*N=2134*	*N=2134*
Prepandemic Group	REF	REF
Pandemic-assessed Group	-2.313 [-3.29, -1.34]**	-2.368 [-3.33, -1.41]**
Pandemic-born Group	-2.958 [-4.39, -1.52]**	-2.723 [-4.16, -1.29]**
**Sample: Leave out PRISM**		
Sample Size	*N=3180*	*N=3180*
Prepandemic Group	REF	REF
Pandemic-assessed Group	-0.806 [-1.59, -0.02]*	-1.084 [-1.85, -0.31]*
Pandemic-born Group	-0.893 [-2.05, 0.26]	-1.017 [-2.17, 0.13]

*p<0.05, **p<0.001
[a]Models include random intercepts for cohort site membership and maternal family.
[b]Models include random intercepts for cohort site membership and maternal family and are adjusted for maternal race (categorical), maternal ethnicity (binary), maternal highest education (categorical), maternal insurance status (binary), child sex (binary), child age at CBCL 1½-5 assessment (continuous), and preterm birth status (binary).
Abbreviations: BAMBAM, Behavior And Mood in Babies And Mothers; BYS, Boricua Youth Study; CI, confidence interval; CIOB, Chemicals in our Bodies; ECHO, Environmental influences on Child Health Outcomes; IKIDS, Illinois Kids Development Study; NYU CHES, New York University Children's Health and Environment Study; PRISM, PRogramming of Intergenerational Stress Mechanisms; PROTECT, ECHO in Puerto Rico.

© 2025 Akbaryan A et al. *JAMA Network Open*.

eTable 3. Leave One Out Sensitivity Analyses Examining the Association Between Timing of Birth and CBCL 1½-5 Assessment Relative to the COVID-19 Pandemic With Child Externalizing Behaviors

	**Model 1[a]** **β[95% CI]**	**Model 2[b]** **β[95% CI]**
**Sample: Leave out PROTECT**		
Sample Size	*N=3024*	*N=3024*
Prepandemic Group	REF	REF
Pandemic-assessed Group	-2.15 [-2.9, -1.4]**	-2.205 [-2.96, -1.45]**
Pandemic-born Group	-3.521 [-4.65, -2.39]**	-3.39 [-4.53, -2.25]**
**Sample: Leave out BAMBAM**		
Sample Size	*N=3275*	*N=3275*
Prepandemic Group	REF	REF
Pandemic-assessed Group	-1.635 [-2.36, -0.91]**	-1.703 [-2.43, -0.97]**
Pandemic-born Group	-2.855 [-3.91, -1.8]**	-2.877 [-3.95, -1.81]**
**Sample: Leave out BYS**		
Sample Size	*N=3182*	*N=3182*
Prepandemic Group	REF	REF
Pandemic-assessed Group	-1.632 [-2.38, -0.89]**	-1.735 [-2.48, -0.99]**
Pandemic-born Group	-3.181 [-4.25, -2.11]**	-3.212 [-4.3, -2.12]**
**Sample: Leave out ECHO Emory**		
Sample Size	*N=3163*	*N=3163*
Prepandemic Group	REF	REF
Pandemic-assessed Group	-1.464 [-2.21, -0.71]**	-1.534 [-2.29, -0.78]**
Pandemic-born Group	-2.937 [-3.99, -1.88]**	-2.926 [-3.99, -1.86]**
**Sample: Leave out Fair Start**		
Sample Size	*N=3345*	*N=3345*
Prepandemic Group	REF	REF
Pandemic-assessed Group	-1.784 [-2.51, -1.06]**	-1.874 [-2.6, -1.15]**
Pandemic-born Group	-3.256 [-4.32, -2.19]**	-3.236 [-4.31, -2.16]**

© 2025 Akbaryan A et al. *JAMA Network Open*.

**Sample: Leave out IKIDS**		
Sample Size	*N=3109*	*N=3109*
Prepandemic Group	REF	REF
Pandemic-assessed Group	-1.82 [-2.58, -1.06]**	-1.885 [-2.65, -1.12]**
Pandemic-born Group	-3.273 [-4.34, -2.2]**	-3.271 [-4.35, -2.19]**
**Sample: Leave out CIOB**		
Sample Size	*N=3092*	*N=3092*
Prepandemic Group	REF	REF
Pandemic-assessed Group	-1.877 [-2.65, -1.1]**	-1.922 [-2.7, -1.15]**
Pandemic-born Group	-3.207 [-4.28, -2.14]**	-3.187 [-4.27, -2.1]**
**Sample: Leave out NYU CHES**		
Sample Size	*N=2134*	*N=2134*
Prepandemic Group	REF	REF
Pandemic-assessed Group	-2.42 [-3.35, -1.49]**	-2.41 [-3.34, -1.48]**
Pandemic-born Group	-4.619 [-5.99, -3.25]**	-4.771 [-6.16, -3.38]**
**Sample: Leave out PRISM**		
Sample Size	*N=3180*	*N=3180*
Prepandemic Group	REF	REF
Pandemic-assessed Group	-1.049 [-1.79, -0.31]*	-1.14 [-1.89, -0.39]*
Pandemic-born Group	-1.989 [-3.08, -0.89]**	-2.1 [-3.21, -0.99]**

*p<0.05, **p<0.001

[a]Models include random intercepts for cohort site membership and maternal family.

[b]Models include random intercepts for cohort site membership and maternal family and are adjusted for maternal race (categorical), maternal ethnicity (binary), maternal highest education (categorical), maternal insurance status (binary), child sex (binary), child age at CBCL 1½-5 assessment (continuous), and preterm birth status (binary).

Abbreviations: BAMBAM, Behavior And Mood in Babies And Mothers; BYS, Boricua Youth Study; CI, confidence interval; CIOB, Chemicals in our Bodies; ECHO, Environmental influences on Child Health Outcomes; IKIDS, Illinois Kids Development Study; NYU CHES, New York University Children's Health and Environment Study; PRISM, PRogramming of Intergenerational Stress Mechanisms; PROTECT, ECHO in Puerto Rico.

© 2025 Akbaryan A et al. *JAMA Network Open*.

eFigure 2. CBCL 1½-5 Assessment Timing Across Cohorts and Exposure Groups

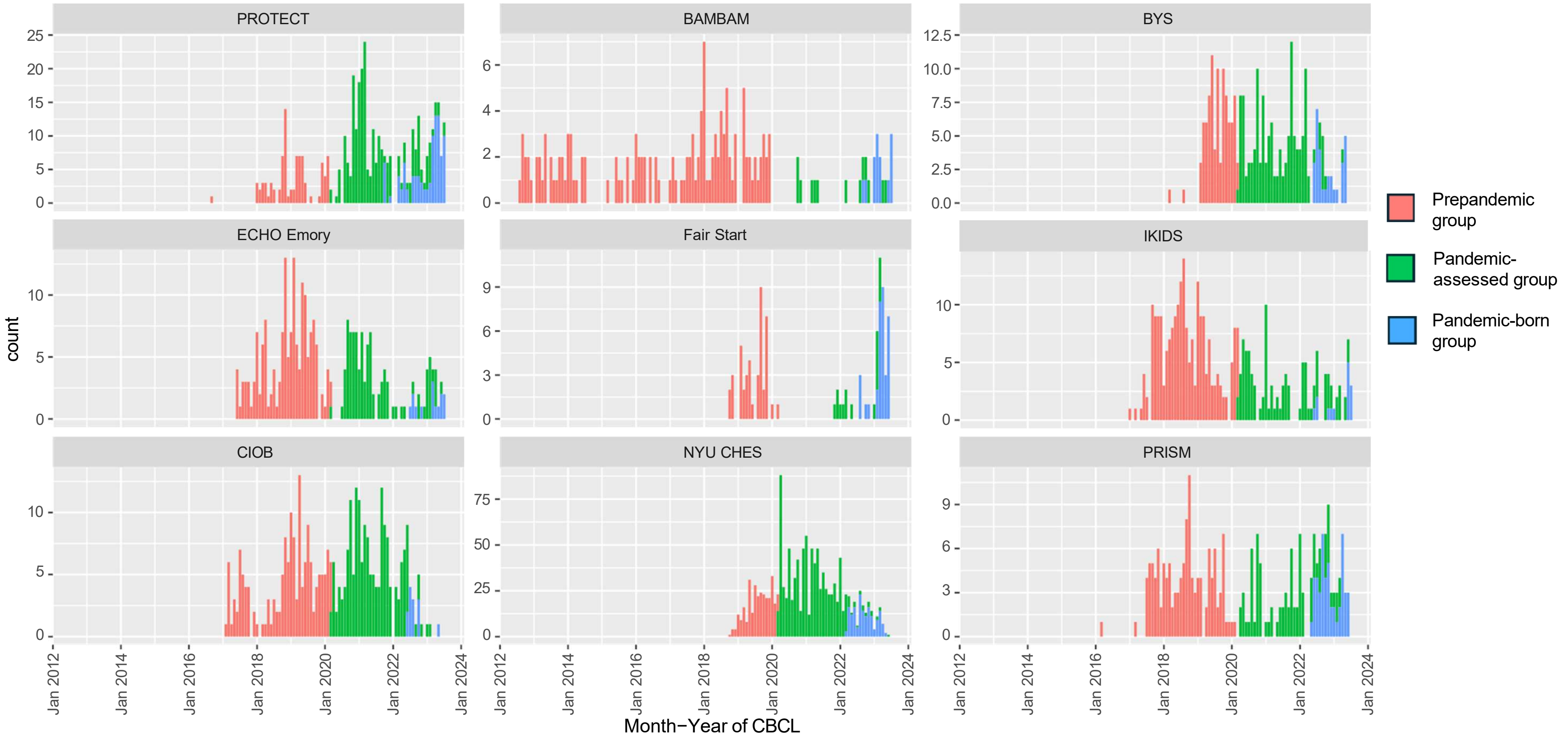

Abbreviations: CBCL, Preschool Child Behavior Checklist 1½-5

© 2025 Akbaryan A et al. *JAMA Network Open*.

eTable 4. Descriptive Table of All CBCL Outcomes Across Exposure Groups

	Prepandemic Group (N=1323)	Pandemic-assessed Group (N=1690)	Pandemic-born Group (N=425)	Overall (N=3438)
**Internalizing problems T-score**				
Mean (SD)	44.12 (10.19)	42.29 (10.05)	42.02 (10.31)	42.96 (10.17)
Median (Min, Max)	43.00 (29.00, 74.00)	41.00 (29.00, 81.00)	41.00 (29.00, 73.00)	43.00 (29.00, 81.00)
Missing	0 (0%)	0 (0%)	0 (0%)	0 (0%)
**Externalizing problems T-score**				
Mean (SD)	45.73 (9.78)	43.71 (9.52)	42.68 (9.65)	44.36 (9.70)
Median (Min, Max)	46.00 (28.00, 79.00)	43.00 (28.00, 77.00)	42.00 (28.00, 73.00)	43.00 (28.00, 79.00)
Missing	0 (0%)	0 (0%)	0 (0%)	0 (0%)
**Total problems T-score**				
Mean (SD)	45.01 (10.21)	43.18 (9.87)	42.53 (10.26)	43.81 (10.09)
Median (Min, Max)	44.00 (28.00, 77.00)	42.00 (28.00, 85.00)	41.00 (28.00, 74.00)	43.00 (28.00, 85.00)
Missing	0 (0%)	0 (0%)	0 (0%)	0 (0%)
**ADHD problems DSM5 T-score**				
Mean (SD)	52.72 (4.66)	52.28 (4.17)	51.89 (4.09)	52.40 (4.37)
Median (Min, Max)	51.00 (50.00, 76.00)	50.00 (50.00, 76.00)	50.00 (50.00, 76.00)	50.00 (50.00, 76.00)
Missing	1 (0.1%)	0 (0%)	2 (0.5%)	3 (0.1%)
**Anxiety problems DSM5 T-score**				
Mean (SD)	52.39 (4.46)	52.17 (4.37)	52.20 (4.39)	52.26 (4.41)
Median (Min, Max)	50.00 (50.00, 84.00)	50.00 (50.00, 84.00)	50.00 (50.00, 75.00)	50.00 (50.00, 84.00)
Missing	1 (0.1%)	4 (0.2%)	2 (0.5%)	7 (0.2%)
**Depressive problems DSM5 T-score**				
Mean (SD)	53.01 (4.79)	52.61 (4.73)	52.76 (4.72)	52.78 (4.75)
Median (Min, Max)	51.00 (50.00, 77.00)	51.00 (50.00, 84.00)	51.00 (50.00, 77.00)	51.00 (50.00, 84.00)
Missing	1 (0.1%)	1 (0.1%)	1 (0.2%)	3 (0.1%)
**Oppositional defiant DSM5 problems T-score**				
Mean (SD)	52.46 (4.67)	51.77 (4.06)	51.83 (4.51)	52.04 (4.37)

© 2025 Akbaryan A et al. *JAMA Network Open*.

Median (Min, Max)	50.00 (50.00, 77.00)	50.00 (50.00, 80.00)	50.00 (50.00, 80.00)	50.00 (50.00, 80.00)
Missing	1 (0.1%)	3 (0.2%)	0 (0%)	4 (0.1%)
**Aggressive Behavior T-score**				
Mean (SD)	52.37 (4.57)	51.72 (3.99)	51.59 (3.98)	51.96 (4.24)
Median (Min, Max)	50.00 (50.00, 79.00)	50.00 (50.00, 79.00)	50.00 (50.00, 79.00)	50.00 (50.00, 79.00)
Missing	0 (0%)	0 (0%)	0 (0%)	0 (0%)
**Anxious/Depressed T-score**				
Mean (SD)	51.85 (3.73)	51.50 (3.31)	51.42 (2.94)	51.63 (3.44)
Median (Min, Max)	50.00 (50.00, 83.00)	50.00 (50.00, 83.00)	50.00 (50.00, 69.00)	50.00 (50.00, 83.00)
Missing	0 (0%)	0 (0%)	0 (0%)	0 (0%)
**Attention problems T-score**				
Mean (SD)	52.96 (4.99)	52.37 (4.20)	52.08 (4.14)	52.56 (4.52)
Median (Min, Max)	51.00 (50.00, 80.00)	50.00 (50.00, 73.00)	50.00 (50.00, 73.00)	51.00 (50.00, 80.00)
Missing	0 (0%)	0 (0%)	0 (0%)	0 (0%)
**Emotionally Reactive T-score**				
Mean (SD)	52.29 (4.37)	51.46 (3.84)	51.28 (3.36)	51.76 (4.02)
Median (Min, Max)	50.00 (50.00, 73.00)	50.00 (50.00, 90.00)	50.00 (50.00, 70.00)	50.00 (50.00, 90.00)
Missing	0 (0%)	2 (0.1%)	1 (0.2%)	3 (0.1%)
**Autism spectrum problems DSM5 T-scores**				
Mean (SD)	53.28 (5.80)	53.10 (6.18)	53.49 (6.58)	53.22 (6.09)
Median (Min, Max)	50.00 (50.00, 81.00)	50.00 (50.00, 98.00)	50.00 (50.00, 91.00)	50.00 (50.00, 98.00)
Missing	3 (0.2%)	5 (0.3%)	0 (0%)	8 (0.2%)
**Sleep problems T-score**				
Mean (SD)	53.30 (5.23)	52.80 (4.92)	52.88 (5.55)	53.00 (5.13)
Median (Min, Max)	51.00 (50.00, 88.00)	50.00 (50.00, 94.00)	50.00 (50.00, 100.00)	51.00 (50.00, 100.00)
Missing	0 (0%)	1 (0.1%)	1 (0.2%)	2 (0.1%)
**Somatic complaints T-score**				
Mean (SD)	52.40 (4.57)	52.15 (4.43)	52.18 (4.82)	52.25 (4.54)
Median (Min, Max)	50.00 (50.00, 74.00)	50.00 (50.00, 80.00)	50.00 (50.00, 80.00)	50.00 (50.00, 80.00)
Missing	2 (0.2%)	1 (0.1%)	0 (0%)	3 (0.1%)

© 2025 Akbaryan A et al. *JAMA Network Open*.

**Withdrawn T-score**				
Mean (SD)	53.20 (5.59)	52.92 (5.93)	53.24 (6.28)	53.07 (5.85)
Median (Min, Max)	51.00 (50.00, 85.00)	50.00 (50.00, 97.00)	50.00 (50.00, 91.00)	50.00 (50.00, 97.00)
Missing	0 (0%)	2 (0.1%)	1 (0.2%)	3 (0.1%)

© 2025 Akbaryan A et al. *JAMA Network Open*.

eResults.

Internalizing Problems by Educational Stratum

In both the "less than Bachelor's degree" stratum and "Bachelor's degree and above" stratum, compared with the Prepandemic Group, children in the Pandemic-assessed Group ("less than Bachelor's degree" β = -2.710, 95% CI: -3.91 to -1.51; "Bachelor's degree and above" β = -1.019, 95% CI: -1.98 to -0.06) and the Pandemic-born Group ("less than Bachelor's degree" β = -3.906, 95% confidence interval [CI]: -5.60 to -2.21; "Bachelor's degree and above" β = -0.380, 95% CI: -1.86 to 1.10) had lower levels of child internalizing T-scores (although one association was not significant). The associations were similar in both strata, but were more pronounced in the "less than Bachelor's degree" group. In both education strata, compared with the Pandemic-assessed Group, children in the Pandemic-born Group ("less than Bachelor's degree" β = -1.230, 95% CI: -2.81 to 0.35; "Bachelor's degree and above" β = 0.64, 95% CI: -0.77 to 2.05) did not differ significantly in child internalizing problem T-scores.

Externalizing Problems by Educational Stratum

In both the "less than Bachelor's degree" stratum and "Bachelor's degree and above" stratum, compared with the Prepandemic Group, children in the Pandemic-assessed Group ("less than Bachelor's degree" β = -2.981, 95% CI: -4.11 to -1.86; "Bachelor's degree and above" β = -0.949, 95% CI: -1.90 to 0.00) and the Pandemic-born Group ("less than Bachelor's degree" β = -5.128, 95% CI: -6.69 to -3.56; "Bachelor's degree and above" β = -2.064, 95% CI: -3.53 to -0.60) had lower externalizing problem T-scores (although one association was marginally significant at alpha level=0.05). The associations were similar in both strata; however, they were more pronounced in the "less than Bachelor's degree" group. Moreover, within the "less than Bachelor's degree" stratum, compared with the Pandemic-assessed Group, children in the Pandemic-born Group ("less than Bachelor's degree" β = -2.147, 95% CI: -3.61 to -0.68; "Bachelor's degree and above" β = -1.113, 95% CI: -2.52 to 0.29) had significantly lower levels of child externalizing T-scores.

eTable 5. CBCL 1½-5 Internalizing and Externalizing *T* Scores Across Assessment Dates and Exposure Groups

CBCL Assessment Timing Periods	Overall	Prepandemic Group All Data	Prepandemic Group Early 2020	Pandemic-assessed Group All Data	Pandemic-assessed Group Early Pandemic	Pandemic-born Group Mid-Late Pandemic (All Data)
CBCL Assessment Dates	**8/14/12–7/21/23**	**8/14/12–3/12/20**	**1/1/20–3/12/20**	**3/13/20–7/18/23**	**3/13/20–6/30/20**	**10/7/21–7/21/23**
**CBCL 1½-5 Internalizing Problems T-Score**						
Mean (SD)	42.96 (10.17)	44.12 (10.19)	43.81 (9.91)	42.29 (10.05)	42.71 (9.32)	42.02 (10.31)
Median [Min, Max]	43 [29, 81]	43 [29, 74]	43 [29, 67]	41 [29, 81]	43 [29, 68]	41 [29, 73]
**CBCL 1½-5 Externalizing Problems T-Score**						
Mean (SD)	44.36 (9.70)	45.73 (9.78)	44.02 (8.48)	43.71 (9.52)	44.55 (9.22)	42.68 (9.65)
Median [Min, Max]	43 [28, 79]	46 [28, 79]	43 [28, 67]	43 [28, 77]	44 [28, 69]	42 [28, 73]

Abbreviations: CBCL 1½-5, Preschool Child Behavior Checklist 1½-5.

eReferences.

1. Van Buuren S, Groothuis-Oudshoorn K. mice: Multivariate imputation by chained equations in R. *Journal of statistical software*. 2011;45:1-67.
2. Heymans MaE, Iris. *Applied missing data analysis with SPSS and (R) Studio*. Heymans and Eekhout: Amsterdam, The Netherlands; 2019.
3. Buuren Sv. *Flexible Imputation of Missing Data, Second Edition*. 2nd Edition ed. Chapman & Hall/CRC Interdisciplinary Statistics. Chapman and Hall/CRC; 2018:444.
4. (FEMA) FEMA. COVID-19 Emergency Declaration. https://www.fema.gov/press-release/20210318/covid-19-emergency-declaration
