# Supplementary material for: COVID-19 Pandemic Exposure and Toddler Behavioral Health in the ECHO Program
Source: JAMA Netw Open. 2025 Sep 3;8(9):e2530346. doi: 10.1001/jamanetworkopen.2025.30346 (PMC12409581; doi:10.1001/jamanetworkopen.2025.30346)
Supplement: Supplement 2. — Nonauthor Collaborators [file jamanetwopen-e2530346-s002.pdf]

Supplemental Online Content: Nonauthor Collaborators
*First name, last name, and suffix (if applicable) are required and will appear in PubMed.

*Group Name(s): ECHO Cohort Consortium							
***First Name and Middle Initial(s)**	***Last Name**	***Suffix (eg, Jr, III)**	Academic Degrees	Institution	Location (city, state/province, country)	Role or Contribution, eg, chair, principal investigator	Group (if more than 1 Group listed in the byline) and/or Subgroup (eg, Steering Committee)
P Brian	Smith		MD, MPH, MHS	Duke Clinical Research Institute, Duke University School of Medicine	Durham, North Carolina, USA	ECHO Coordinating Center Principal Investigator	
L Kristin	Newby		MD, MHS	Duke Clinical Research Institute, Duke University School of Medicine	Durham, North Carolina, USA	ECHO Coordinating Center Principal Investigator	
Linda	Adair		PhD	Gillings School of Global Public Health, University of North Carolina at Chapel Hill	Chapel Hill, North Carolina, USA	ECHO Coordinating Center Principal Investigator	
Lisa P.	Jacobson		ScD	Johns Hopkins University, Bloomberg School of Public Health	Baltimore, Maryland, USA	ECHO Data Analysis Center Principal Investigator	
Diane	Catellier		DrPH	Research Triangle Institute	Research Triangle Park, North Carolina, USA	ECHO Data Analysis Center Principal Investigator	
Monica	McGrath		ScD	Johns Hopkins University, Bloomberg School of Public Health	Baltimore, Maryland, USA	ECHO Johns Hopkins University Data Analysis Center Director Co-Investigator	
Christian	Douglas		DrPH	Research Triangle Institute	Research Triangle Park, North Carolina, USA	ECHO RTI Data Analysis Center Director Co-Investigator	
Priya	Duggal		PhD	Johns Hopkins University, Bloomberg School of Public Health	Baltimore, Maryland, USA	ECHO Data Analysis Center Genetics Methods Lead, Co-Investigator	

Supplemental Online Content: Nonauthor Collaborators
*First name, last name, and suffix (if applicable) are required and will appear in PubMed.

***First Name and Middle Initial(s)**	***Last Name**	***Suffix (eg, Jr, III)**	Academic Degrees	Institution	Location (city, state/province, country)	Role or Contribution, eg, chair, principal investigator	Group (if more than 1 Group listed in the byline) and/or Subgroup (eg, Steering Committee)
Emily	Knapp		PhD	Johns Hopkins University, Bloomberg School of Public Health	Baltimore, Maryland, USA	ECHO Data Analysis Center Co-Investigator	
Amii	Kress		PhD	Johns Hopkins University, Bloomberg School of Public Health	Baltimore, Maryland, USA	ECHO Data Analysis Center General Methods Co-Investigator	
Courtney K.	Blackwell		PhD	Feinberg School of Medicine, Northwestern University	Chicago, Illinois, USA	Measurement Core Co-Investigator	
Maxwell A.	Mansolf		PhD	Feinberg School of Medicine, Northwestern University	Chicago, Illinois, USA	Measurement Core Co-Investigator	
Jin-Shei	Lai		PhD	Feinberg School of Medicine, Northwestern University	Chicago, Illinois, USA	Measurement Core Co-Investigator	
Emily	Ho		PhD	Feinberg School of Medicine, Northwestern University	Chicago, Illinois, USA	Measurement Core Co-Investigator	
David	Cella		PhD	Feinberg School of Medicine, Northwestern University	Chicago, Illinois, USA	Measurement Core Principal Investigator	
Richard	Gershon		PhD	Feinberg School of Medicine, Northwestern University	Chicago, Illinois, USA	Measurement Core Principal Investigator	
Michelle L.	Macy		MD	Feinberg School of Medicine, Northwestern University and Ann & Robert H. Lurie Children's Hospital of Chicago	Chicago, Illinois, USA	Measurement Core Co-Investigator	
Suman R.	Das		PhD	Vanderbilt University Medical Center	Nashville, Tennessee, USA	ECHO Laboratory Core Principal Investigator	
Jane E.	Freedman		MD	Vanderbilt University Medical Center	Nashville, Tennessee, USA	ECHO Laboratory Core Principal Investigator	

*First name, last name, and suffix (if applicable) are required and will appear in PubMed.

***First Name and Middle Initial(s)**	***Last Name**	***Suffix (eg, Jr, III)**	Academic Degrees	Institution	Location (city, state/province, country)	Role or Contribution, eg, chair, principal investigator	Group (if more than 1 Group listed in the byline) and/or Subgroup (eg, Steering Committee)
Simon A.	Mallal		MBBS	Vanderbilt University Medical Center	Nashville, Tennessee, USA	ECHO Laboratory Core Principal Investigator	
John A.	McLean		PhD	Vanderbilt University	Nashville, Tennessee, USA	ECHO Laboratory Core Principal Investigator	
Ravi V.	Shah		MD	Vanderbilt University Medical Center	Nashville, Tennessee, USA	ECHO Laboratory Core Principal Investigator	
Meghan H.	Shilts		MHS	Vanderbilt University Medical Center	Nashville, Tennessee, USA	ECHO Laboratory Core Principal Investigator Admin Designee	
Akram N.	Alshawabkeh		PhD	Northeastern University	Boston, Massachusetts, USA	ECHO Cohort Study Site Principal Investigator	
Jose F.	Cordero		MD	University of Georgia	Athens, Georgia; USA	ECHO Cohort Study Site Co-Director	
John	Meeker		ScD	University of Michigan	Ann Arbor, Michigan; USA	ECHO Cohort Study Site Co-Director	
Leonardo	Trasande		MD, MPP	NYU Grossman School of Medicine	New York, New York, USA	ECHO Cohort Study Site Principal Investigator	
Carlos A.	Camargo	Jr.	MD, DrPH	Massachusetts General Hospital, Harvard Medical School	Boston, Massachusetts, USA	ECHO Cohort Study Site Principal Investigator	
Kohei	Hasegawa		MD, PhD	Massachusetts General Hospital, Harvard Medical School	Boston, Massachusetts, USA	ECHO Cohort Study Site Co-Investigator	
Zhaozhong	Zhu		ScD	Massachusetts General Hospital, Harvard Medical School	Boston, Massachusetts, USA	ECHO Cohort Study Site Co-Investigator	

Supplemental Online Content: Nonauthor Collaborators
*First name, last name, and suffix (if applicable) are required and will appear in PubMed.

***First Name and Middle Initial(s)**	***Last Name**	***Suffix (eg, Jr, III)**	Academic Degrees	Institution	Location (city, state/province, country)	Role or Contribution, eg, chair, principal investigator	Group (if more than 1 Group listed in the byline) and/or Subgroup (eg, Steering Committee)
Ashley F.	Sullivan		MS, MPH	Massachusetts General Hospital, Harvard Medical School	Boston, Massachusetts, USA	ECHO Cohort Study Site Award Project Director	
Dana	Dabelea		MD, PhD	University of Colorado Anschutz Medical Campus	Aurora, Colorado, USA	ECHO Cohort Study Site Principal Investigator	
Wei	Perng		PhD, MPH	University of Colorado Anschutz Medical Campus	Aurora, Colorado, USA	ECHO Cohort Study Site Principal Investigator	
Traci A.	Bekelman		PhD, MPH	University of Colorado Anschutz Medical Campus	Aurora, Colorado, USA	ECHO Cohort Study Site Principal Investigator	
Greta	Wilkening		PhD, MPH	University of Colorado Anschutz Medical Campus	Aurora, Colorado, USA	ECHO Cohort Study Site Co-Investigator	
Sheryl	Magzamen		PhD	Colorado School of Public Health, Colorado State University	Fort Collins, Colorado, USA	ECHO Cohort Study Site Co-Investigator	
Brianna F.	Moore		PhD, MS	University of Colorado Anschutz Medical Campus	Aurora, Colorado, USA	ECHO Cohort Study Site Principal Investigator	
Anne P.	Starling		PhD	University of North Carolina at Chapel Hill	Chapel Hill, North Carolina, USA	ECHO Cohort Study Site Principal Investigator	
Deborah J.	Rinehart		PhD	Denver Health and Hospital Authority	Denver, Colorado, USA	ECHO Cohort Study Site Co-Investigator	
Daphne	Koinis Mitchell		Ph.D	Rhode Island Hospital, The Alpert Medical School of Brown University	Providence, Rhode Island, USA	ECHO Cohort Study Site Principal Investigator	
Viren	D'Sa		MD	Rhode Island Hospital, The Alpert Medical School of Brown University	Providence, Rhode Island, USA	ECHO Cohort Study Site Principal Investigator	

*First name, last name, and suffix (if applicable) are required and will appear in PubMed.

***First Name and Middle Initial(s)**	***Last Name**	***Suffix (eg, Jr, III)**	Academic Degrees	Institution	Location (city, state/province, country)	Role or Contribution, eg, chair, principal investigator	Group (if more than 1 Group listed in the byline) and/or Subgroup (eg, Steering Committee)
Sean C.L.	Deoni		PhD	Bill & Melinda Gates Foundation	Seattle, Washington, USA	ECHO Cohort Study Site Principal Investigator	
Hans-Georg	Mueller		PhD	University of California, Davis	Davis, California, USA	ECHO Cohort Study Site Co-Investigator	
Cristiane S.	Duarte		PhD, MPH	Columbia University - NYSPI	New York, New York, USA	ECHO Cohort Study Site Principal Investigator	
Catherine	Monk		PhD	Columbia University - NYSPI	New York, New York, USA	ECHO Cohort Study Site Principal Investigator	
Glorisa	Canino		PhD	University of Puerto Rico, School of Medicine	Rio Piedras, Puerto Rico	ECHO Cohort Study Site Principal Investigator	
Jonathan	Posner		MD	Duke University School of Medicine, Duke Psychiatry & Behavioral Sciences	Durham, North Carolina, USA	ECHO Cohort Study Site Principal Investigator	
Tenneill	Murray		MPH	Columbia University - NYSPI	New York, New York, USA	ECHO Cohort Study Site Co-Director	
Claudia	Lugo-Candelas		PhD	Columbia University - NYSPI	New York, New York, USA	ECHO Cohort Study Site Principal Investigator	
Anne L.	Dunlop		MD, MPH	Emory University School of Medicine	Atlanta, Georgia, USA	ECHO Cohort Study Site Principal Investigator	
Patricia A.	Brennan		PhD	Emory University	Atlanta, Georgia, USA	ECHO Cohort Study Site Principal Investigator	
Christine	Hockett		PhD	Avera Research Institute; University of South Dakota School of Medicine	Rapid City, South Dakota, USA; Sioux Falls, South Dakota, USA	ECHO Cohort Study Site Principal Investigator	

Supplemental Online Content: Nonauthor Collaborators
*First name, last name, and suffix (if applicable) are required and will appear in PubMed.

***First Name and Middle Initial(s)**	***Last Name**	***Suffix (eg, Jr, III)**	Academic Degrees	Institution	Location (city, state/province, country)	Role or Contribution, eg, chair, principal investigator	Group (if more than 1 Group listed in the byline) and/or Subgroup (eg, Steering Committee)
Amy	Elliott		PhD	Avera Research Institute ; University of South Dakota School of Medicine	Sioux Falls, South Dakota, USA	ECHO Cohort Study Site Principal Investigator	
Assiamira	Ferrara		MD, PhD	Kaiser Permanente Northern California	Oakland, California, USA	ECHO Cohort Study Site Principal Investigator	
Lisa A.	Croen		PhD	Kaiser Permanente Northern California	Oakland, California, USA	ECHO Cohort Study Site Principal Investigator	
Monique M.	Hedderson		PhD	Kaiser Permanente Northern California	Oakland, California, USA	ECHO Cohort Study Site Principal Investigator	
John	Ainsworth		PhD	University of Manchester	Manchester, United Kingdom	ECHO Cohort Study Site Principal Investigator	
Leonard B.	Bacharier		MD	Vanderbilt University Medical Center	Nashville, Tennessee, USA	ECHO Cohort Study Site Principal Investigator	
Casper G.	Bendixsen		PhD	Marshfield Clinic Research Institute	Marshfield, Wisconsin, USA	ECHO Cohort Study Site Principal Investigator	
James E.	Gern		MD	University of Wisconsin School of Medicine and Public Health	Madison, Wisconsin, USA	ECHO Cohort Study Site Principal Investigator	
Diane R.	Gold		MD	Brigham and Women’s Hospital; Harvard Medical School	Boston, Massachusetts, USA	ECHO Cohort Study Site Principal Investigator	
Tina V.	Hartert		MD, MPH	Vanderbilt University Medical Center	Nashville, Tennessee, USA	ECHO Cohort Study Site Principal Investigator	

Supplemental Online Content: Nonauthor Collaborators
*First name, last name, and suffix (if applicable) are required and will appear in PubMed.

***First Name and Middle Initial(s)**	***Last Name**	***Suffix (eg, Jr, III)**	Academic Degrees	Institution	Location (city, state/province, country)	Role or Contribution, eg, chair, principal investigator	Group (if more than 1 Group listed in the byline) and/or Subgroup (eg, Steering Committee)
Daniel J.	Jackson		MD	University of Wisconsin School of Medicine and Public Health	Madison, Wisconsin, USA	ECHO Cohort Study Site Principal Investigator	
Christine C.	Johnson		PhD	Henry Ford Health	Detroit, Michigan, USA	ECHO Cohort Study Site Principal Investigator	
Christine L.M.	Joseph		PhD	Henry Ford Health	Detroit, Michigan, USA	ECHO Cohort Study Site Principal Investigator	
Meyer	Kattan		MD	Columbia University Medical Center	New York, New York, USA	ECHO Cohort Study Site Principal Investigator	
Gurjit K.	Khurana Hershey		MD, PhD	Cincinnati Children's Hospital Medical Center	Cincinnati, Ohio, USA	ECHO Cohort Study Site Principal Investigator	
Robert F.	Lemanske, Jr.		MD	University of Wisconsin School of Medicine and Public Health	Madison, Wisconsin, USA	ECHO Cohort Study Site Principal Investigator	
Susan V.	Lynch		PhD	University of California	San Francisco, California, USA	ECHO Cohort Study Site Principal Investigator	
Rachel L.	Miller		MD	Icahn School of Medicine at Mount Sinai	New York, New York, USA	ECHO Cohort Study Site Principal Investigator	
George T.	O'Connor		MD	Boston University School of Medicine	Boston, Massachusetts, USA	ECHO Cohort Study Site Principal Investigator	
Carole	Ober		PhD	University of Chicago	Chicago, Illinois, USA	ECHO Cohort Study Site Principal Investigator	

*First name, last name, and suffix (if applicable) are required and will appear in PubMed.

***First Name and Middle Initial(s)**	***Last Name**	***Suffix (eg, Jr, III)**	Academic Degrees	Institution	Location (city, state/province, country)	Role or Contribution, eg, chair, principal investigator	Group (if more than 1 Group listed in the byline) and/or Subgroup (eg, Steering Committee)
Dennis	Ownby		MD	Henry Ford Health	Detroit, Michigan, USA	ECHO Cohort Study Site Principal Investigator	
Katherine	Rivera-Spoljaric		MD	Washington University School of Medicine	St Louis, Missouri, USA	ECHO Cohort Study Site Principal Investigator	
Patrick H.	Ryan		PhD	University of Cincinnati	Cincinnati, Ohio, USA	ECHO Cohort Study Site Principal Investigator	
Christine M.	Seroogy		MD	University of Wisconsin School of Medicine and Public Health	Madison, Wisconsin, USA	ECHO Cohort Study Site Principal Investigator	
Anne Marie	Singh		MD	University of Wisconsin School of Medicine and Public Health	Madison, Wisconsin, USA	ECHO Cohort Study Site Principal Investigator	
Robert A.	Wood		MD	Johns Hopkins University School of Medicine	Baltimore, Maryland, USA	ECHO Cohort Study Site Principal Investigator	
Edward M.	Zoratti		MD	Henry Ford Health	Detroit, Michigan, USA	ECHO Cohort Study Site Principal Investigator	
Rima	Habre		ScD, MSc	University of Southern California	Los Angeles, California, USA	ECHO Cohort Study Site Principal Investigator	
Shohreh	Farzan		PhD	University of Southern California	Los Angeles, California, USA	ECHO Cohort Study Site Principal Investigator	
Frank D.	Gilliland		MD, MPH, PhD	University of Southern California	Los Angeles, California, USA	ECHO Cohort Study Site Principal Investigator	

*First name, last name, and suffix (if applicable) are required and will appear in PubMed.

***First Name and Middle Initial(s)**	***Last Name**	***Suffix (eg, Jr, III)**	Academic Degrees	Institution	Location (city, state/province, country)	Role or Contribution, eg, chair, principal investigator	Group (if more than 1 Group listed in the byline) and/or Subgroup (eg, Steering Committee)
Irva	Hertz-Picciotto		PhD	University of California, Davis	Davis, California, USA	ECHO Cohort Study Site Principal Investigator	
Deborah H.	Bennett		Ph.D	University of California, Davis	Davis, California, USA	ECHO Cohort Study Site Principal Investigator	
Julie B.	Schweitzer		Ph.D	University of California, Davis	Davis, California, USA	ECHO Cohort Study Site Principal Investigator	
Rebecca J.	Schmidt		Ph.D	University of California, Davis	Davis, California, USA	ECHO Cohort Study Site Principal Investigator	
Janine M.	LaSalle		PhD	University of California, Davis	Davis, California, USA	ECHO Cohort Study Site Co-Investigator	
Alison E.	Hipwell		PhD, ClinPsyD	University of Pittsburgh	Pittsburgh, Pennsylvania, USA	ECHO Cohort Study Site Principal Investigator	
Catherine J.	Karr		MD, MS, PhD	University of Washington	Seattle, Washington, USA	ECHO Cohort Study Site Principal Investigator	
Nicole R.	Bush		PhD	University of California, San Francisco	San Francisco, California, USA	ECHO Cohort Study Site Principal Investigator	
Kaja Z.	LeWinn		ScD	University of California, San Francisco	San Francisco, California, USA	ECHO Cohort Study Site Principal Investigator	
Sheela	Sathyanarayana		MD, MPH	University of Washington and Seattle Children's Research Institute	Seattle, Washington, USA	ECHO Cohort Study Site Principal Investigator	

Supplemental Online Content: Nonauthor Collaborators
*First name, last name, and suffix (if applicable) are required and will appear in PubMed.

***First Name and Middle Initial(s)**	***Last Name**	***Suffix (eg, Jr, III)**	Academic Degrees	Institution	Location (city, state/province, country)	Role or Contribution, eg, chair, principal investigator	Group (if more than 1 Group listed in the byline) and/or Subgroup (eg, Steering Committee)
Qi	Zhao		MD, PhD	University of Tennessee Health Science Center	Memphis, Tennessee, USA	ECHO Cohort Study Site Principal Investigator	
Frances	Tylavsky		DrPH, MS	University of Tennessee Health Science Center	Memphis, Tennessee, USA	ECHO Cohort Study Site Principal Investigator	
Kecia N.	Carroll		MD, MPH	Icahn School of Medicine at Mount Sinai	New York, New York, USA	ECHO Cohort Study Site Principal Investigator	
Christine T.	Loftus		MS MPH PhD	University of Washington	Seattle, Washington, USA	ECHO Cohort Study Site Principal Investigator	
Leslie D.	Leve		PhD	University of Oregon	Eugene, Oregon, USA	ECHO Cohort Study Site Principal Investigator	
Jody M.	Ganiban		PhD	George Washington University	Washington, DC, USA	ECHO Cohort Study Site Principal Investigator	
Jenae M.	Neiderhiser		PhD	Penn State University	University Park, Pennsylvania, USA	ECHO Cohort Study Site Principal Investigator	
Scott T.	Weiss		MD	Brigham and Women's Hospital and Harvard Medical School	Boston, Massachusetts, USA	ECHO Cohort Study Site Principal Investigator	
Augusto A.	Litonjua		MD	Golisano Children's Hospital, University of Rochester	Rochester, New York, USA	ECHO Cohort Study Site Principal Investigator	
Cindy T.	McEvoy		MD, MCR	Oregon Health & Science University	Portland, Oregon, USA	ECHO Cohort Study Site Principal Investigator	

Supplemental Online Content: Nonauthor Collaborators
*First name, last name, and suffix (if applicable) are required and will appear in PubMed.

***First Name and Middle Initial(s)**	***Last Name**	***Suffix (eg, Jr, III)**	Academic Degrees	Institution	Location (city, state/province, country)	Role or Contribution, eg, chair, principal investigator	Group (if more than 1 Group listed in the byline) and/or Subgroup (eg, Steering Committee)
Eliot R.	Spindel		MD, PhD	Oregon National Primate Research Center	Beaverton, Oregon, USA	ECHO Cohort Study Site Principal Investigator	
Robert S.	Tepper		MD, PhD	Indiana School of Medicine	Indianapolis, Indiana, USA	ECHO Cohort Study Site Co-Investigator	
Craig J.	Newschaffer		PhD	Penn State	State College, Pennsylvania, USA	ECHO Cohort Study Site Principal Investigator	
Kristen	Lyall		ScD	Drexel University	Philadelphia, Pennsylvania, USA	ECHO Cohort Study Site Principal Investigator	
Heather E.	Volk		PhD	Johns Hopkins University	Baltimore, Maryland, USA	ECHO Cohort Study Site Principal Investigator	
Rebecca	Landa		PhD	Center for Autism and Related Disorders, Kennedy Krieger Institute, Johns Hopkins University	Baltimore, Maryland, USA	ECHO Cohort Study Site Co-Investigator	
Sally	Ozonoff		PhD	University of California Davis	Sacramento, California, USA	ECHO Cohort Study Site Co-Investigator	
Joseph	Piven		MD	University of North Carolina	Chapel Hill, North Carolina, USA	ECHO Cohort Study Site Co-Investigator	
Heather	Hazlett		PhD	University of North Carolina	Chapel Hill, North Carolina, USA	ECHO Cohort Study Site Co-Investigator	
Juhi	Pandey		PhD	Children's Hospital of Philadelphia	Philadelphia, Pennsylvania, USA	ECHO Cohort Study Site Co-Investigator	
Robert	Schultz		PhD	Children's Hospital of Philadelphia	Philadelphia, Pennsylvania, USA	ECHO Cohort Study Site Co-Investigator	
Steven	Dager		PhD	University of Washington	Seattle, Washington, USA	ECHO Cohort Study Site Co-Investigator	

Supplemental Online Content: Nonauthor Collaborators
*First name, last name, and suffix (if applicable) are required and will appear in PubMed.

***First Name and Middle Initial(s)**	***Last Name**	***Suffix (eg, Jr, III)**	Academic Degrees	Institution	Location (city, state/province, country)	Role or Contribution, eg, chair, principal investigator	Group (if more than 1 Group listed in the byline) and/or Subgroup (eg, Steering Committee)
Kelly	Botteron		PhD	Washington University	St Louis, Missouri, USA	ECHO Cohort Study Site Co-Investigator	
Daniel	Messinger		PhD	University of Miami	Miami, Florida, USA	ECHO Cohort Study Site Co-Investigator	
Wendy	Stone		PhD	University of Washington	Seattle, Washington, USA	ECHO Cohort Study Site Co-Investigator	
Jennifer	Ames		PhD	Kaiser Permanente	Oakland, California, USA	ECHO Cohort Study Site Co-Investigator	
Thomas G.	O'Connor		PhD	University of Rochester	Rochester, New York, USA	ECHO Cohort Study Site Principal Investigator	
Richard K.	Miller		PhD	University of Rochester	Rochester, New York, USA	ECHO Cohort Study Site Principal Investigator	
Emily	Oken		MD, MPH	Harvard Pilgrim Health Care Institute and Harvard Medical School	Boston, Massachusetts, USA	ECHO Cohort Study Site Principal Investigator	
Michele R.	Hacker		ScD	Beth Israel Deaconess Medical Center	Boston, Massachusetts, USA	ECHO Cohort Study Site Principal Investigator	
Tamarra	James-Todd		PhD	Harvard Chan School of Public Health	Boston, Massachusetts, USA	ECHO Cohort Study Site Principal Investigator	
T. Michael	O'Shea	Jr	MD, MPH	University of North Carolina School of Medicine	Chapel Hill, North Carolina, USA	ECHO Cohort Study Site Principal Investigator	
Rebecca C.	Fry		PhD	University of North Carolina Gillings School of Global Public Health	Chapel Hill, North Carolina, USA	ECHO Cohort Study Site Principal Investigator	
Jean A.	Frazier		MD	UMASS Chan Medical School	Worcster, Massachusetts, USA	ECHO Cohort Study Site Co-Investigator	

Supplemental Online Content: Nonauthor Collaborators
*First name, last name, and suffix (if applicable) are required and will appear in PubMed.

***First Name and Middle Initial(s)**	***Last Name**	***Suffix (eg, Jr, III)**	Academic Degrees	Institution	Location (city, state/province, country)	Role or Contribution, eg, chair, principal investigator	Group (if more than 1 Group listed in the byline) and/or Subgroup (eg, Steering Committee)
Rachana	Singh		MD, MS	Tufts University School of Medicine	Boston, Massachusetts, USA	ECHO Cohort Study Site Co-Investigator	
Caitlin	Rollins		MD, SM	Harvard Medical School	Boston, Massachusetts, USA	ECHO Cohort Study Site Co-Investigator	
Angela	Montgomery		MD	Yale School of Medicine	New Haven, Connecticut, USA	ECHO Cohort Study Site Co-Investigator	
Ruben	Vaidya		MD	University of Massachusetts Chan Medical School-Baystate	Springfield, Massachusetts, USA	ECHO Cohort Study Site Co-Investigator	
Robert M.	Joseph		PhD	Boston University Chobanian & Avedisian School of Medicine	Boston, Massachusetts, USA	ECHO Cohort Study Site Co-Investigator	
Lisa K.	Washburn		MD	Wake Forest School of Medicine	Winston-Salem, North Carolina, USA	ECHO Cohort Study Site Co-Investigator	
Semsa	Gogcu		MD, MPH	Wake Forest School of Medicine; Wake Forest University School of Medicine/Atrium Health Wake Forest	Winston-Salem, North Carolina, USA	ECHO Cohort Study Site Co-Investigator	
Kelly	Bear		DO	ECU Health	Greenville, North Carolina, USA	ECHO Cohort Study Site Co-Investigator	
Julie V.	Rollins		MA	University of North Carolina School of Medicine	Chapel Hill, North Carolina, USA	ECHO Cohort Study Site Award Project Director	
Stephen R.	Hooper		PhD	School of Medicine, University of North Carolina at Chapel Hill	Chapel Hill, North Carolina, USA	ECHO Cohort Study Site Co-Investigator	
Genevieve	Taylor		MD	School of Medicine, University of North Carolina at Chapel Hill	Chapel Hill, North Carolina, USA	ECHO Cohort Study Site Co-Investigator	
Wesley	Jackson		MD, MPH	University of North Carolina School of Medicine	Chapel Hill, North Carolina, USA	ECHO Cohort Study Site Co-Investigator	
Amanda	Thompson		PhD	University of North Carolina at Chapel Hill; Gillings School of Global Public Health, University of North Carolina at Chapel Hill	Chapel Hill, North Carolina, USA	ECHO Cohort Study Site Co-Investigator	

Supplemental Online Content: Nonauthor Collaborators
*First name, last name, and suffix (if applicable) are required and will appear in PubMed.

***First Name and Middle Initial(s)**	***Last Name**	***Suffix (eg, Jr, III)**	Academic Degrees	Institution	Location (city, state/province, country)	Role or Contribution, eg, chair, principal investigator	Group (if more than 1 Group listed in the byline) and/or Subgroup (eg, Steering Committee)
Julie	Daniels		PhD	University of North Carolina at Chapel Hill; Gillings School of Global Public Health, University of North Carolina at Chapel Hill	Chapel Hill, North Carolina, USA	ECHO Cohort Study Site Co-Investigator	
Michelle	Hernandez		MD	School of Medicine, University of North Carolina at Chapel Hill	Chapel Hill, North Carolina, USA	ECHO Cohort Study Site Co-Investigator	
Kun	Lu		PhD	Gillings School of Global Public Health, University of North Carolina at Chapel Hill	Chapel Hill, North Carolina, USA	ECHO Cohort Study Site Co-Investigator	
Michael	Msall		MD	University of Chicago Medicine: Comer Children's Hospital	Chicago Illinois, USA	ECHO Cohort Study Site Co-Investigator	
Madeleine	Lenski		MSPH	Michigan State University	East Lansing, Michigan, USA	ECHO Cohort Study Site Co-Investigator	
Rawad	Obeid		MD	Beaumont Hospital	Royal Oak, Michigan, USA	ECHO Cohort Study Site Co-Investigator	
Steven L.	Pastyrnak		PhD	Corewell Health, Helen DeVos Children's Hospital	Grand Rapids, Michigan, USA	ECHO Cohort Study Site Co-Investigator	
Elizabeth	Jensen		PhD	Wake Forest University School of Medicine	Winston-Salem, North Carolina, USA	ECHO Cohort Study Site Co-Investigator	
Christina	Sakai		MD	Mass General Hospital for Children	Boston, Massachusetts, USA	ECHO Cohort Study Site Co-Investigator	
Hudson	Santos		RN, PhD	University of Miami	Coral Gables, Florida, USA	ECHO Cohort Study Site Principal Investigator	
Jean M.	Kerver		PhD, MSc, RD	Michigan State University, College of Human Medicine	East Lansing, Michigan, USA	ECHO Cohort Study Site Principal Investigator	
Nigel	Paneth		MD, MPH	Michigan State University, College of Human Medicine	East Lansing, Michigan, USA	ECHO Cohort Study Site Principal Investigator	

Supplemental Online Content: Nonauthor Collaborators
*First name, last name, and suffix (if applicable) are required and will appear in PubMed.

***First Name and Middle Initial(s)**	***Last Name**	***Suffix (eg, Jr, III)**	Academic Degrees	Institution	Location (city, state/province, country)	Role or Contribution, eg, chair, principal investigator	Group (if more than 1 Group listed in the byline) and/or Subgroup (eg, Steering Committee)
Charles J.	Barone	II	MD, FAAP	Henry Ford Health	Detroit, Michigan, USA	ECHO Cohort Study Site Principal Investigator	
Michael R.	Elliott		PhD	University of Michigan	Ann Arbor, Michigan, USA	ECHO Cohort Study Site Principal Investigator	
Douglas M.	Ruden		PhD	Wayne State University	Detroit, Michigan, USA	ECHO Cohort Study Site Principal Investigator	
Chris	Fussman		MS	Michigan Department of Health and Human Services (MDHHS)	Lansing, Michigan, USA	ECHO Cohort Study Site Principal Investigator	
Julie B.	Herbstman		PhD	Columbia University Mailman School of Public Health	New York, New York, USA	ECHO Cohort Study Site Principal Investigator	
Amy	Margolis		PhD	Columbia University Irving Medical Center	New York, New York, USA	ECHO Cohort Study Site Principal Investigator	
Susan L.	Schantz		PhD	University of Illinois Urbana-Champaign	Urbana, Illinois, USA	ECHO Cohort Study Site Principal Investigator	
Sarah Dee	Geiger		PhD	University of Illinois Urbana-Champaign	Urbana, Illinois, USA	ECHO Cohort Study Site Co-Investigator	
Andrea	Aguiar		PhD	University of Illinois Urbana-Champaign	Urbana, Illinois, USA	ECHO Cohort Study Site Co-Investigator	
Karen	Tabb		PhD, MSW	University of Illinois Urbana-Champaign	Urbana, Illinois, USA	ECHO Cohort Study Site Co-Investigator	
Rita	Strakovsky		PhD	Michigan State University	East Lansing, Michigan, USA	ECHO Cohort Study Site Co-Investigator	

Supplemental Online Content: Nonauthor Collaborators
*First name, last name, and suffix (if applicable) are required and will appear in PubMed.

***First Name and Middle Initial(s)**	***Last Name**	***Suffix (eg, Jr, III)**	Academic Degrees	Institution	Location (city, state/province, country)	Role or Contribution, eg, chair, principal investigator	Group (if more than 1 Group listed in the byline) and/or Subgroup (eg, Steering Committee)
Tracey	Woodruff		PhD, MPH	University of California, San Francisco	San Francisco, California, USA	ECHO Cohort Study Site Principal Investigator	
Rachel	Morello-Frosch		PhD, MPH	University of California, Berkeley	Berkeley, California, USA	ECHO Cohort Study Site Principal Investigator	
Amy	Padula		PhD	University of California, San Francisco	San Francisco, California, USA	ECHO Cohort Study Site Co-Investigator	
Joseph B.	Stanford		MD, MSPH	Spencer Fox Eccles School of Medicine, University of Utah	Salt Lake City, Utah, USA	ECHO Cohort Study Site Principal Investigator	
Christina A.	Porucznik		PhD, MSPH	Spencer Fox Eccles School of Medicine, University of Utah	Salt Lake City, Utah, USA	ECHO Cohort Study Site Principal Investigator	
Angelo P.	Giardino		MD, PhD	Spencer Fox Eccles School of Medicine, University of Utah	Salt Lake City, Utah, USA	ECHO Cohort Study Site Principal Investigator	
Rosalind J.	Wright		MD, MPH	Icahn School of Medicine at Mount Sinai	New York, New York, USA	ECHO Cohort Study Site Principal Investigator	
Robert O.	Wright		MD, MPH	Icahn School of Medicine at Mount Sinai	New York, New York, USA	ECHO Cohort Study Site Principal Investigator	
Brent	Collett		PhD	University of Washington, Seattle Children's Research Institute	Seattle, Washington, USA	ECHO Cohort Study Site Principal Investigator	
Nicole	Baumann-Blackmore		MD	University of Wisconsin School of Medicine and Public Health	Madison, Wisconsin, USA	ECHO Cohort Study Site Co-Investigator	
Ronald	Gangnon		PhD	University of Wisconsin	Madison, Wisconsin, USA	ECHO Cohort Study Site Co-Investigator	

Supplemental Online Content: Nonauthor Collaborators
*First name, last name, and suffix (if applicable) are required and will appear in PubMed.

***First Name and Middle Initial(s)**	***Last Name**	***Suffix (eg, Jr, III)**	Academic Degrees	Institution	Location (city, state/province, country)	Role or Contribution, eg, chair, principal investigator	Group (if more than 1 Group listed in the byline) and/or Subgroup (eg, Steering Committee)
Daniel J.	Jackson		MD	University of Wisconsin School of Medicine and Public Health	Madison, Wisconsin, USA	ECHO Cohort Study Site Co-Investigator	
Chris G.	McKennan		PhD	University of Pittsburgh	Pittsburgh, Pennsylvania, USA	ECHO Cohort Study Site Co-Investigator	
Jo	Wilson		MD	University of Wisconsin School of Medicine and Public Health	Madison, Wisconsin, USA	ECHO Cohort Study Site Co-Investigator	
Matt	Altman		MD	University of Washington	Seattle, Washington, USA	ECHO Cohort Study Site Co-Investigator	
Judy L.	Aschner		MD	Albert Einstein College of Medicine; Hackensack Meridian School of Medicine; Center for Discovery and Innovation	Bronx, New York, USA; Nutley, New Jersey, USA	ECHO Cohort Study Site Principal Investigator	
Annemarie	Stroustrup		MD, MPH	Northwell Health, Cohen Children's Medical Center, and the Zucker School of Medicine at Hofstra / Northwell	New Hyde Park, New York, USA	ECHO Cohort Study Site Principal Investigator	
Stephanie L.	Merhar		MD, MS	Cincinnati Children's	Cincinnati, Ohio, USA	ECHO Cohort Study Site Co-Investigator	
Paul E.	Moore		MD	Vanderbilt University Medical Center	Nashville, Tennessee, USA	ECHO Cohort Study Site Co-Investigator	
Gloria S.	Pryhuber		MD	University of Rochester Medical Center	Rochester, New York, USA	ECHO Cohort Study Site Co-Investigator	
Mark	Hudak		MD	University of Florida College of Medicine	Jacksonville, Florida, USA	ECHO Cohort Study Site Co-Investigator	
Ann Marie	Reynolds Lyndaker		MD, MPH	University of Buffalo Jacobs School of Medicine and Biomedical Sciences	Buffalo, New York, USA	ECHO Cohort Study Site Co-Investigator	
Andrea L.	Lampland		MD	Children's Minnesota	Minneapolis, Minnesota, USA	ECHO Cohort Study Site Co-Investigator	

Supplemental Online Content: Nonauthor Collaborators
*First name, last name, and suffix (if applicable) are required and will appear in PubMed.

***First Name and Middle Initial(s)**	***Last Name**	***Suffix (eg, Jr, III)**	Academic Degrees	Institution	Location (city, state/province, country)	Role or Contribution, eg, chair, principal investigator	Group (if more than 1 Group listed in the byline) and/or Subgroup (eg, Steering Committee)
Burton	Rochelson		MD	Northwell Health and the Zucker School of Medicine at Hofstra / Northwell	New Hyde Park, New York, USA	ECHO Cohort Study Site Principal Investigator	
Sophia	Jan		MD, MSHP	Northwell Health, Cohen Children's Medical Center, and the Zucker School of Medicine at Hofstra / Northwell	New Hyde Park, New York, USA	ECHO Cohort Study Site Co-Investigator	
Matthew J.	Blitz		MD, MBA	Northwell Health and the Zucker School of Medicine at Hofstra / Northwell	New Hyde Park, New York, USA	ECHO Cohort Study Site Co-Investigator	
Michelle W.	Katzow		MD, MS	Northwell Health, Cohen Children's Medical Center, and the Zucker School of Medicine at Hofstra / Northwell	New Hyde Park, New York, USA	ECHO Cohort Study Site Co-Investigator	
Zenobia	Brown		MD, MPH	Northwell Health and the Zucker School of Medicine at Hofstra / Northwell	New Hyde Park, New York, USA	ECHO Cohort Study Site Co-Investigator	
Codruta	Chiuzan		PhD	Northwell Health, Feinstein Institutes for Medical Research	Manhasset, New York, USA	ECHO Cohort Study Site Co-Investigator	
Timothy	Rafael		MD	Northwell Health and the Zucker School of Medicine at Hofstra / Northwell	New Hyde Park, New York, USA	ECHO Cohort Study Site Co-Investigator	
Dawnette	Lewis		MD, MPH	Northwell Health and the Zucker School of Medicine at Hofstra / Northwell	New Hyde Park, New York, USA	ECHO Cohort Study Site Co-Investigator	
Natalie	Meirowitz		MD	Northwell Health and the Zucker School of Medicine at Hofstra / Northwell	New Hyde Park, New York, USA	ECHO Cohort Study Site Co-Investigator	
Brenda	Poindexter		MD	Children's Healthcare of Atlanta Emory University	Atlanta, Georgia, USA	ECHO Cohort Study Site Co-Investigator	

Supplemental Online Content: Nonauthor Collaborators
*First name, last name, and suffix (if applicable) are required and will appear in PubMed.

***First Name and Middle Initial(s)**	***Last Name**	***Suffix (eg, Jr, III)**	Academic Degrees	Institution	Location (city, state/province, country)	Role or Contribution, eg, chair, principal investigator	Group (if more than 1 Group listed in the byline) and/or Subgroup (eg, Steering Committee)
Tebeb	Gebretsadik		MPH	Vanderbilt University Medical Center	Nashville, Tennessee, USA	ECHO Cohort Study Site Principal Investigator	
Sarah	Osmundson		MD, MSC	Vanderbilt University Medical Center	Nashville, Tennessee, USA	ECHO Cohort Study Site Principal Investigator	
Jennifer K.	Straughen		PhD	Henry Ford Health	Detroit, Michigan, USA	ECHO Cohort Study Site Principal Investigator	
Amy	Eapen		MD	Henry Ford Health	Detroit, Michigan, USA	ECHO Cohort Study Site Principal Investigator	
Andrea	Cassidy-Bushrow		PhD	Henry Ford Health	Detroit, Michigan, USA	ECHO Cohort Study Site Co-Investigator	
Ganesa	Wegienka		PhD	Henry Ford Health	Detroit, Michigan, USA	ECHO Cohort Study Site Co-Investigator	
Alex	Sitarik		MPH	Henry Ford Health	Detroit, Michigan, USA	ECHO Cohort Study Site Biostatistician	
Kim	Woodcroft		PhD	Henry Ford Health	Detroit, Michigan, USA	ECHO Cohort Study Site Co-Investigator	
Audrey	Urquhart		MPH	Henry Ford Health	Detroit, Michigan, USA	ECHO Cohort Study Site Epidemiologist	
Albert	Levin		PhD	Henry Ford Health	Detroit, Michigan, USA	ECHO Cohort Study Site Co-Investigator	
Tisa	Johnson-Hooper		MD	Henry Ford Health	Detroit, Michigan, USA	ECHO Cohort Study Site Co-Investigator	
Brent	Davidson		MD	Henry Ford Health	Detroit, Michigan, USA	ECHO Cohort Study Site Co-Investigator	
Tengfei	Ma		PhD	Henry Ford Health	Detroit, Michigan, USA	ECHO Cohort Study Site Co-Investigator	

Supplemental Online Content: Nonauthor Collaborators
*First name, last name, and suffix (if applicable) are required and will appear in PubMed.

*First Name and Middle Initial(s)	*Last Name	*Suffix (eg, Jr, III)	Academic Degrees	Institution	Location (city, state/province, country)	Role or Contribution, eg, chair, principal investigator	Group (if more than 1 Group listed in the byline) and/or Subgroup (eg, Steering Committee)
Emily S.	Barrett		PhD	Environmental and Occupational Health Sciences Institute, Rutgers University	Piscataway, New Jersey, USA	ECHO Cohort Study Site Principal Investigator	
Martin J.	Blaser		MD	Rutgers University	Piscataway, New Jersey, USA	ECHO Cohort Study Site Principal Investigator	
Maria Gloria	Dominguez-Bello		PhD	Rutgers University	New Brunswick, New Jersey, USA	ECHO Cohort Study Site Principal Investigator	
Daniel B.	Horton		MD	Robert Wood Johnson Medical School, Rutgers University	New Brunswick, New Jersey, USA	ECHO Cohort Study Site Principal Investigator	
Manuel	Jimenez		MD	Robert Wood Johnson Medical School, Rutgers University	New Brunswick, New Jersey, USA	ECHO Cohort Study Site Principal Investigator	
Todd	Rosen		MD	Robert Wood Johnson Medical School, Rutgers University	New Brunswick, New Jersey, USA	ECHO Cohort Study Site Co-Investigator	
Kristy	Palomares		MD, PhD	Saint Peter's University Hospital	New Brunswick, New Jersey, USA	ECHO Cohort Study Site Co-Investigator	
Lyndsay A.	Avalos		PhD, MPH	Kaiser Permanente Northern California	Oakland, California, USA	ECHO Cohort Study Site Principal Investigator	
Yeyi	Zhu		PhD, MS	Kaiser Permanente Northern California	Oakland, California, USA	ECHO Cohort Study Site Principal Investigator	
Kelly J .	Hunt		PhD	Medical University of South Carolina	Charleston, South Carolina, USA	ECHO Cohort Study Site Principal Investigator	
Roger B.	Newman		MD	Medical University of South Carolina	Charleston, South Carolina, USA	ECHO Cohort Study Site Principal Investigator	

Supplemental Online Content: Nonauthor Collaborators
*First name, last name, and suffix (if applicable) are required and will appear in PubMed.

***First Name and Middle Initial(s)**	***Last Name**	***Suffix (eg, Jr, III)**	Academic Degrees	Institution	Location (city, state/province, country)	Role or Contribution, eg, chair, principal investigator	Group (if more than 1 Group listed in the byline) and/or Subgroup (eg, Steering Committee)
Michael S.	Bloom		PhD	George Mason University	Fairfax, Virginia, USA	ECHO Cohort Study Site Principal Investigator	
Mallory H.	Alkis		MD	Medical University of South Carolina	Charleston, South Carolina, USA	ECHO Cohort Study Site Co-Investigator	
James R.	Roberts		MD, MPH	Medical University of South Carolina	Charleston, South Carolina, USA	ECHO Cohort Study Site Co-Investigator	
Sunni L.	Mumford		PhD	University of Pennsylvania Perelman School of Medicine	Philadelphia, Pennsylvania, USA	ECHO Cohort Study Site Principal Investigator	
Heather H.	Burris		MD, MPH	Children's Hospital of Philadelphia; University of Pennsylvania Perelman School of Medicine	Philadelphia, Pennsylvania, USA	ECHO Cohort Study Site Principal Investigator	
Sara B.	DeMauro		MD, MSCE	Children's Hospital of Philadelphia; University of Pennsylvania Perelman School of Medicine	Philadelphia, Pennsylvania, USA	ECHO Cohort Study Site Principal Investigator	
Lynn M.	Yee		MD, MPH	Feinberg School of Medicine, Northwestern University	Chicago, Illinois, USA	ECHO Cohort Study Site Principal Investigator	
Aaron	Hamvas		MD	Ann & Robert H. Lurie Children's Hospital, Feinberg School of Medicine, Northwestern University	Chicago, Illinois, USA	ECHO Cohort Study Site Principal Investigator	
Antonia F.	Olidipo		MD, MSCI	Hackensack University Medical Center, Hackensack Meridian School of Medicine	Nutley, New Jersey, USA	ECHO Cohort Study Site Co-Investigator	
Andrew S.	Haddad		MD	Hackensack University Medical Center, Hackensack Meridian School of Medicine	Nutley, New Jersey, USA	ECHO Cohort Study Site Co-Investigator	

Supplemental Online Content: Nonauthor Collaborators
*First name, last name, and suffix (if applicable) are required and will appear in PubMed.

***First Name and Middle Initial(s)**	***Last Name**	***Suffix (eg, Jr, III)**	Academic Degrees	Institution	Location (city, state/province, country)	Role or Contribution, eg, chair, principal investigator	Group (if more than 1 Group listed in the byline) and/or Subgroup (eg, Steering Committee)
Lisa R.	Eiland		MD	Hackensack University Medical Center, Hackensack Meridian School of Medicine	Nutley, New Jersey, USA	ECHO Cohort Study Site Co-Investigator	
Nicole T.	Spillane		MD	Hackensack University Medical Center, Hackensack Meridian School of Medicine	Nutley, New Jersey, USA	ECHO Cohort Study Site Co-Investigator	
Kirin N.	Suri		MD	Hackensack University Medical Center, Hackensack Meridian School of Medicine	Nutley, New Jersey, USA	ECHO Cohort Study Site Co-Investigator	
Stephanie A.	Fisher		MD, MPH	Feinberg School of Medicine, Northwestern University	Chicago, Illinois, USA	ECHO Cohort Study Site Co-Investigator	
Jeffrey A.	Goldstein		MD, PhD	Feinberg School of Medicine, Northwestern University	Chicago, Illinois, USA	ECHO Cohort Study Site Co-Investigator	
Leena B.	Mithal		MD	Ann & Robert H. Lurie Children's Hospital, Feinberg School of Medicine, Northwestern University	Chicago, Illinois, USA	ECHO Cohort Study Site Co-Investigator	
Raye-Ann O.	DeRegnier		MD	Ann & Robert H. Lurie Children's Hospital, Feinberg School of Medicine, Northwestern University	Chicago, Illinois, USA	ECHO Cohort Study Site Co-Investigator	
Nathalie L.	Maitre		MD, PhD	Emory University School of Medicine and Cerebral Palsy Foundation	Atlanta, Georgia, USA and New York, New York, USA	ECHO Cohort Study Site Co-Investigator	
Ruby H.N.	Nguyen		PhD, MHS	School of Public Health, University of Minnesota	Minneapolis, Minnesota, USA	ECHO award Principal Investigator	
Meghan M.	JaKa		PhD, MS	HealthPartners Institute	Minneapolis, Minnesota, USA	ECHO site Principal Investigator	
Abbey C.	Sidebottom		PhD, MPH	Allina Health	Minneapolis, Minnesota, USA	ECHO site Principal Investigator	

Supplemental Online Content: Nonauthor Collaborators
*First name, last name, and suffix (if applicable) are required and will appear in PubMed.

***First Name and Middle Initial(s)**	***Last Name**	***Suffix (eg, Jr, III)**	Academic Degrees	Institution	Location (city, state/province, country)	Role or Contribution, eg, chair, principal investigator	Group (if more than 1 Group listed in the byline) and/or Subgroup (eg, Steering Committee)
Michael J.	Paidas		MD	University of Miami Miller School of Medicine	Miami, Florida, USA	ECHO site Principal Investigator	
JoNell E.	Potter		APRN, PhD	University of Miami Miller School of Medicine	Miami, Florida, USA	ECHO Cohort Study Site Co-Investigator	
Natale	Ruby		PhD, PsyD	University of Miami Miller School of Medicine	Miami, Florida, USA	ECHO Cohort Study Site Co-Investigator	
Lunthita	Duthely		EdD	University of Miami School of Medicine	Miami, Florida, USA	ECHO Cohort Study Site Co-Investigator	
Arumugam	Jayakumar		PhD	University of Miami Miller School of Medicine	Miami, Florida, USA	ECHO Cohort Study Site Co-Investigator	
Karen	Young		MD	University of Miami Miller School of Medicine	Miami, Florida, USA	ECHO Cohort Study Site Co-Investigator	
Isabel	Maldonado		MPH, BS	University of Miami	Miami, Florida, USA	ECHO Cohort Study Site Program Director	
Meghan	Miller		PhD	University of California Davis	Sacramento, California, USA	ECHO Cohort Study Site Co-Investigator	
Jonathan L.	Slaughter		MD, MPH	Nationwide Children's Hospital and The Ohio State University	Columbus, Ohio, USA	ECHO Cohort Study Site Principal Investigator	
Sarah A.	Keim		PhD, MS, MA	Nationwide Children's Hospital and The Ohio State University	Columbus, Ohio, USA	ECHO Cohort Study Site Principal Investigator	
Courtney D.	Lynch		PhD, MPH	The Ohio State University	Columbus, Ohio, USA	ECHO Cohort Study Site Principal Investigator	
Kartik K.	Venkatesh		MD, PhD	The Ohio State University	Columbus, Ohio, USA	ECHO Cohort Study Site Principal Investigator	

Supplemental Online Content: Nonauthor Collaborators
*First name, last name, and suffix (if applicable) are required and will appear in PubMed.

*First Name and Middle Initial(s)	*Last Name	*Suffix (eg, Jr, III)	Academic Degrees	Institution	Location (city, state/province, country)	Role or Contribution, eg, chair, principal investigator	Group (if more than 1 Group listed in the byline) and/or Subgroup (eg, Steering Committee)
Kristina W.	Whitworth		PhD	Baylor College of Medicine	Houston, Texas, USA	ECHO Cohort Study Site Principal Investigator	
Elaine	Symanski		PhD	Baylor College of Medicine	Houston, Texas, USA	ECHO Cohort Study Site Principal Investigator	
Thomas F.	Northrup		PhD	University of Texas Health Science Center at Houston (UTHealth Houston) McGovern Medical School	Houston, Texas, USA	ECHO Cohort Study Site Principal Investigator	
Hector	Mendez-Figueroa		MD	University of Texas Health Science Center at Houston (UTHealth Houston) McGovern Medical School	Houston, Texas, USA	ECHO Cohort Study Site Co-Investigator	
Ricardo A.	Mosquera		MD	University of Texas Health Science Center at Houston (UTHealth Houston) McGovern Medical School	Houston, Texas, USA	ECHO Cohort Study Site Co-Investigator	
Margaret R.	Karagas		PhD	Geisel School of Medicine at Dartmouth	Hanover, New Hampshire, USA	ECHO Cohort Study Site Principal Investigator	
Juliette C.	Madan		MD, MS	Geisel School of Medicine at Dartmouth, Dartmouth Hitchcock Medical Center	Hanover, New Hampshire, USA	ECHO Cohort Study Site Principal Investigator	
Debra M.	MacKenzie		PhD	College of Pharmacy, University of New Mexico Health Sciences Center	Albuquerque, New Mexico, USA	ECHO Cohort Study Site Principal Investigator	
Johnnye L.	Lewis		PhD	College of Pharmacy, University of New Mexico Health Sciences Center	Albuquerque, New Mexico, USA	ECHO Cohort Study Site Principal Investigator	

Supplemental Online Content: Nonauthor Collaborators
*First name, last name, and suffix (if applicable) are required and will appear in PubMed.

***First Name and Middle Initial(s)**	***Last Name**	***Suffix (eg, Jr, III)**	Academic Degrees	Institution	Location (city, state/province, country)	Role or Contribution, eg, chair, principal investigator	Group (if more than 1 Group listed in the byline) and/or Subgroup (eg, Steering Committee)
Brandon J.	Rennie		PhD	University of New Mexico	Albuquerque, New Mexico, USA	ECHO Cohort Study Site Co-Investigator	
Bennett L.	Leventhal		MD	College of Pharmacy, University of New Mexico Health Sciences Center; University of Chicago	Albuquerque, New Mexico, USA; Chicago, Illinois, USA	ECHO Cohort Study Site Co-Investigator	
Young Shin	Kim		MD, MS, MPH, PhD	University of California, San Francisco	San Francisco, California, USA	ECHO Cohort Study Site Co-Investigator	
Somer	Bishop		PhD	University of California, San Francisco	San Francisco, California, USA	ECHO Cohort Study Site Co-Investigator	
Sara S.	Nozadi		PhD	College of Pharmacy, University of New Mexico Health Sciences Center	Albuquerque, New Mexico, USA	ECHO Cohort Study Site Co-Investigator	
Li	Luo		PhD	Comprehensive Cancer Center, University of New Mexico Health Sciences Center	Albuquerque, New Mexico, USA	ECHO Cohort Study Site Co-Investigator	
Barry M.	Lester		PhD	Warren Alpert Medical School of Brown University	Providence, Rhode Island, USA	ECHO Cohort Study Site Principal Investigator	
Carmen J.	Marsit		PhD	Rollins School of Public Health, Emory University	Atlanta, Georgia, USA	ECHO Cohort Study Site Principal Investigator	
Todd	Everson		PhD	Rollins School of Public Health, Emory University	Atlanta, Georgia, USA	ECHO Cohort Study Site Principal Investigator	
Cynthia M.	Loncar		PhD	Warren Alpert Medical School of Brown University	Providence, Rhode Island, USA	ECHO Cohort Study Site Principal Investigator	
Elisabeth C.	McGowan		MD	Warren Alpert Medical School of Brown University	Providence, Rhode Island, USA	ECHO Cohort Study Site Principal Investigator	

Supplemental Online Content: Nonauthor Collaborators
*First name, last name, and suffix (if applicable) are required and will appear in PubMed.

***First Name and Middle Initial(s)**	***Last Name**	***Suffix (eg, Jr, III)**	Academic Degrees	Institution	Location (city, state/province, country)	Role or Contribution, eg, chair, principal investigator	Group (if more than 1 Group listed in the byline) and/or Subgroup (eg, Steering Committee)
Stephen J.	Sheinkopf		PhD	Thompson Center for Autism & Neurodevelopment, University of Missouri	Columbia, Missouri, USA	ECHO Cohort Study Site Principal Investigator	
Brian S.	Carter		MD	Children's Mercy-Kansas City	Kansas City, Missouri, USA	ECHO Cohort Study Site Principal Investigator	
Jennifer	Check		MD	Wake Forest School of Medicine	Winston, Salem North Carolina, USA	ECHO Cohort Study Site Principal Investigator	
Jennifer B.	Helderman		MD	Wake Forest School of Medicine	Winston, Salem North Carolina, USA	ECHO Cohort Study Site Principal Investigator	
Charles R.	Neal		MD	University of Hawaii John A Burns School of Medicine	Honolulu, Hawaii, USA	ECHO Cohort Study Site Principal Investigator	
Lynne M.	Smith		MD	UCLA Clinical and Translational Science Institute at The Lundquist Institute, Harbor-UCLA Medical Center	Los Angeles, California, USA	ECHO Cohort Study Site Principal Investigator	
