# Supplementary material for: COVID-19 Pandemic Exposure and Toddler Behavioral Health in the ECHO Program
Source: JAMA Netw Open. 2025 Sep 3;8(9):e2530346. doi: 10.1001/jamanetworkopen.2025.30346 (PMC12409581; doi:10.1001/jamanetworkopen.2025.30346)
Supplement: Supplement 3. — Data Sharing Statement [file jamanetwopen-e2530346-s003.pdf]

Data Sharing Statement

Akbaryan. COVID-19 Pandemic Exposure and Toddler Behavioral Health in the ECHO Program. *JAMA Netw Open*. Published September 03, 2025.
doi:10.1001/jamanetworkopen.2025.30346

Data
Data available: Yes
Data types: Deidentified participant data
How to access data: Select de-identified data from the ECHO Program are available through NICHD's Data and Specimen Hub (DASH) (https://dash.nichd.nih.gov/). Information on study data not available on DASH, such as some Indigenous datasets, can be found on the ECHO study DASH webpage (https://dash.nichd.nih.gov/explore/study?q=echo&filters=%5b%5d&page=1&sortBy=relevance&asc=true&size=50).
When available: With publication

Supporting Documents
Document types: None

Additional Information
Who can access the data: anyone requesting the data
Types of analyses: for any purpose
Mechanisms of data availability: Data are publicly available.
